# 24-hour Serum Creatinine Variation Associates with Short- and Long-Term All-Cause Mortality: A Real-World Insight into Early Detection of Acute Kidney Injury

**DOI:** 10.1038/s41598-020-63315-x

**Published:** 2020-04-16

**Authors:** Hung-Chieh Yeh, Yen-Chun Lo, I-Wen Ting, Pei-Lun Chu, Shih-Ni Chang, Hsiu-Yin Chiang, Chin-Chi Kuo

**Affiliations:** 1 AKI-CARE (Clinical Advancement, Research and Education) Center, Department of Internal Medicine, China Medical University Hospital and College of Medicine, China Medical University, Taichung, Taiwan; 2 Division of Nephrology, Department of Internal Medicine, China Medical University Hospital and College of Medicine, China Medical University, Taichung, Taiwan; 3 Big Data Center, China Medical University Hospital and College of Medicine, China Medical University, Taichung, Taiwan; 4 Division of Nephrology, Department of Internal Medicine, Fu Jen Catholic University Hospital, and School of Medicine, College of Medicine, Fu Jen Catholic University, New Taipei City, Taiwan

**Keywords:** Biomarkers, Nephrology

## Abstract

Real-world evidence describing the variation in serum creatinine (S-Cre) within 24 hours and its prognostic value is unknown. We enrolled 14 912 adults who received two S-Cre measurements within 24 hours at a tertiary hospital between 2003 and 2016. The study population was divided into four groups according to the hospital service settings where the baseline and second S-Cre were measured: Group 1, Outpatient-to-Outpatient; Group 2, Outpatient-to-ED (emergency department) or Inpatient; Group 3, ED-to-ED or Inpatient; and Group 4, Inpatient-to-Inpatient. The main predictors were the difference between the two S-Cre measurements (ΔS-Cre) and the percent change (ΔS-Cre%). The main outcomes were 30-day, 1-year, or 3-year all-cause mortality. A total of 6753 and 8159 patients with an increase and a decrease within-day ΔS-Cre, respectively. Among 6753 patients who had deteriorating ΔS-Cre or ΔS-Cre%, the adjusted hazard ratio (aHR) for 1-year all-cause mortality for each 0.1 mg/dL or 5% change in S-Cre was 1.09 (95% confidence interval [CI]: 1.07, 1.11) and 1.03 (95% CI: 1.03, 1.04). In 8159 patients with improving ΔS-Cre%, the aHR was 0.97 (95% CI: 0.94, 1.00). Groups 3 and 4 had statistically significant positive linear relationships between deteriorating ΔS-Cre% and 30-day and 3-year mortality. The optimal cut-offs for deteriorating ΔS-Cre% for predicting 30-day mortality were approximately 22% for Group 3 and 20% for Group 4. Inpatient within-day deteriorating ΔS-Cre or ΔS-Cre% above 0.2 mg/dL or 20%, respectively, is associated with all-cause mortality. Monitoring 24-hour S-Cre variation identifies acute kidney injury earlier than the conventional criteria.

## Introduction

The prognostic importance of serum creatinine (S-Cre) within-day variation has not been widely evaluated. In clinical practice, critical variations, defined by an absolute increase of 0.3 mg/dL (26.5 μmol/L) in S-Cre within 48 hours or a 50% increase in S-Cre concentration over a 7-day period, have been used to diagnose in-hospital acute kidney injury (AKI) since 2004^1,2^. A 100% increase in S-Cre concentration is a conventional study endpoint in chronic kidney disease (CKD) research, despite a recent study proposing the less stringent criterion of 30%, with the aim to capture more clinical outcomes while retaining prognostic significance^3^. However, these cut-offs are arbitrary because of the lack of detailed knowledge, obtained from real-world evidence, regarding the within-day variation in people with a wide range of kidney function.

The diurnal fluctuation in S-Cre, mainly due to alimentary factors, was first observed 50 years ago. Among healthy participants, research has revealed that the maximum mean within-day percent change in S-Cre is almost 30%^4^. The main sources of within-day S-Cre variation include analytical and biological within-subject variations. Much effort has focused on controlling the analytical variation of inter-assay variability or inherent measurement error by establishing calibration traceability using isotope dilution mass spectrometry (IDMS) reference procedures^5^. Yet, our understanding of the biological variation in S-Cre remains confined to factors that can influence levels of S-Cre such as the individual’s muscle mass, renal tubular secretion, protein-rich intake, and extra-renal clearance of creatinine through intestinal microbes^6–8^. More importantly, claiming that S-Cre variation is physiological or pathological without linking an individual’s survival outcome to the within-day variation of S-Cre would be far from evidence-based. To clarify the clinical meaning of within-day variation in S-Cre, we tracked the mortality of patients who had received repeated S-Cre measurements within 24 hours.

## Results

A total of 6753 patients with an increase in within-day ΔS-Cre and 8159 patients with a decrease contributed a total of 24 257 and 31 400 person-years of follow-up, respectively (Supplementary Table 1a,b). The median age on the index day was 60.4 (IQR: 46.6, 74.2) years for patients with deteriorating ΔS-Cre and 59.0 (44.8, 73.3) years for patients with improving ΔS-Cre. Patients in the lowest quartile of ΔS-Cre% were older and more likely to be male compared with those in the three highest quartiles (Supplementary Table 1a,b). For comorbidities, the prevalence of impaired kidney function (IKF), acute kidney failure (AKF), diabetes, and hypertension decreased across the quartiles of deteriorating ΔS-Cre% (Supplementary Table 1a), but similar comorbidity trends were also observed among patients with improving ΔS-Cre% (Supplementary Table 1b). The increased use of nonsteroidal anti-inflammatory drugs (NSAIDs) and radiocontrast was observed across the increasing quartiles of improving and deteriorating ΔS-Cre%. The baseline white blood cell counts (WBC) and C-reactive protein (CRP) increased across the increasing quartiles of ΔS-Cre%, and the potassium and serum albumin levels decreased (Supplementary Table 1a,b). Corresponding clinical characteristics of ΔS-Cre, by quartiles, are provided in Supplementary Table 2a,b showing similar results.

**Figure 1:**
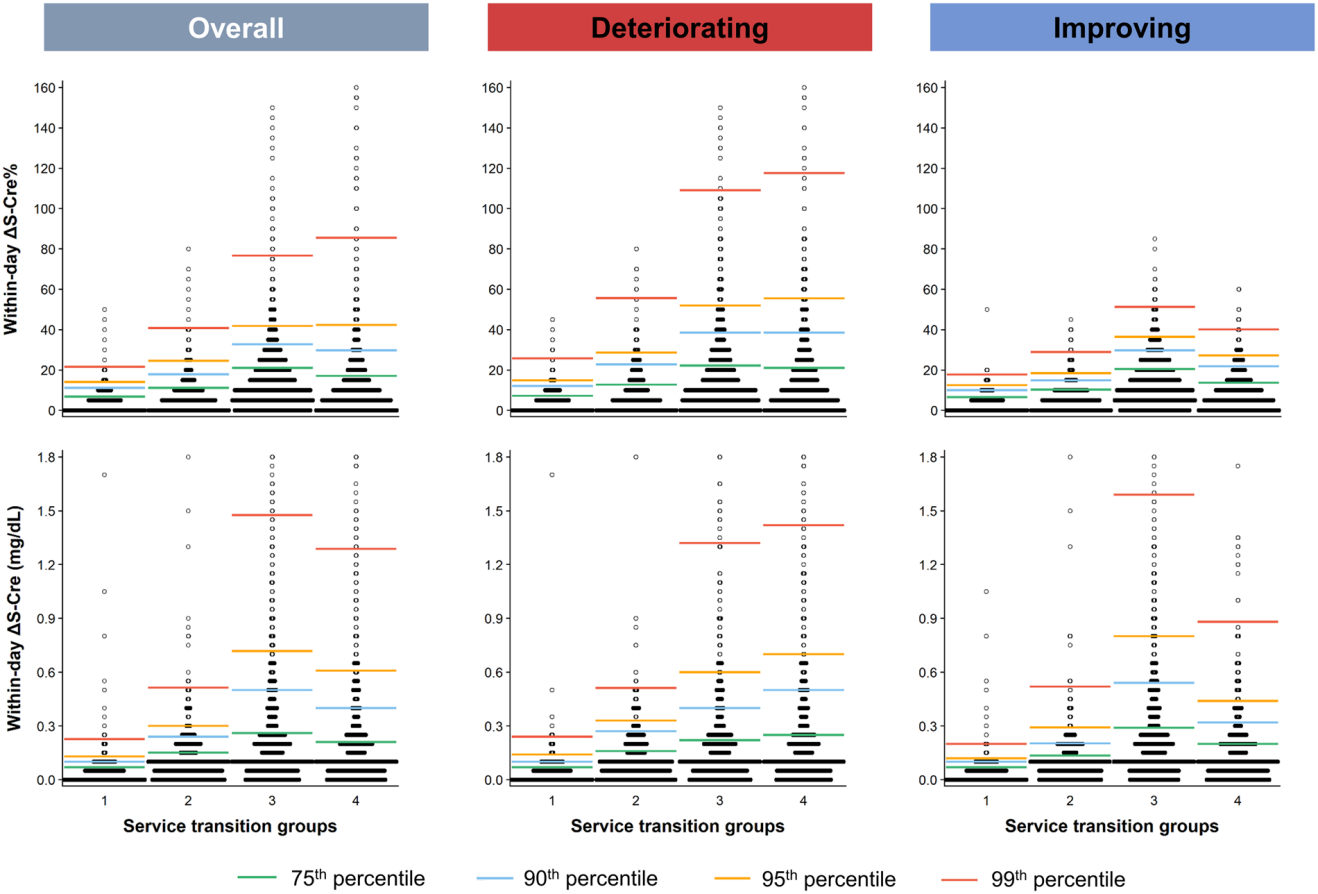
Box-percentile plot of the difference and percent change in S-Cre levels within 24 hours (within-day ΔS-Cre and ΔS-Cre%) in overall population and in patients with deteriorating or improving kidney function based on patients’ service transition patterns (Four groups: Group 1, OPT-to-OPT; Group 2, OPT-to-ED or INPT; Group 3, ED-to-ED or INPT; and Group 4, INPT–to-INPT). Specific percentiles are highlighted by color lines: median, green; 75^th^ percentile, blue; 90^th^ percentile, yellow; and 95^th^ percentile, red. ED, emergency department; INPT, inpatient; OPT, outpatient; S-Cre, serum creatinine.

From the perspective of the four service transition groups, patients in Group 4 (INPT-to-INPT) were older; more likely to be male; had the highest prevalence of IKF and noncancerous catastrophic status; were more likely to be exposed to diuretics, NSAIDs, and radiocontrast; and had developed the highest all-cause mortality during the follow-up (Table 1). The proportion of patients who received intravenous fluid therapy on the index day was up to 97.2% for Group 3 and 82.6% for Group 4. Group 4 also had the highest baseline levels of blood urea nitrogen (BUN), S-Cre, serum sodium, CRP, and WBC and had the lowest baseline levels of serum albumin and hemoglobin (Table 1). Figure 1 summarizes the distribution of ΔS-Cre and ΔS-Cre% in the present study population by the four service transition groups.

**Table 1:** Baseline demographic and clinical characteristics based on patients’ service transition patterns: ED, emergency department; INPT, inpatient; OPT, outpatient.

Variables	Group 1	Group 2	Group 3	Group 4	*p*-value^a^
	OPT to OPT	OPT to ED or INPT	ED to ED or INPT	INPT to INPT	
**Demographics, median (IQR)**	4145 (27.8%)	1761 (11.8%)	5545 (37.2%)	**3164 (21.2%)**	
Age, years	58.7 (47.3, 70.5)	64.0 (50.2, 75.6)	56.7 (40.4, 73.2)	64.3 (49.7, 77.5)	<0.001
Men, n (%)	2481 (59.9)	951 (54.0)	3317 (59.8)	1894 (59.9)	0.418
Body mass index (kg/m^2^)	24.7 (22.2, 27.6)	23.8 (21.2, 26.8)	23.5 (20.8, 26.5)	23.5 (20.6, 26.6)	<0.001
**Comorbidities, n (%)**					
Impaired kidney function	873 (21.1)	666 (37.8)	1641 (29.6)	1243 (39.3)	<0.001
Acute kidney failure (ICD 584.5–584.9)	100 (2.41)	138 (7.84)	412 (7.43)	246 (7.77)	<0.001
Diabetes mellitus	1195 (28.8)	599 (34.0)	935 (16.9)	997 (31.5)	<0.001
Hypertension	1418 (34.2)	686 (39.0)	983 (17.7)	1017 (32.1)	<0.001
Noncancerous Catastrophic illness status	356 (8.59)	229 (13.00)	1058 (19.08)	768 (24.27)	<0.001
**Therapy, n (%)**					
Fluid therapy between two measurements	29 (0.70)	853 (48.44)	5015 (90.44)	2060 (65.11)	<0.001
Fluid therapy on the index day	527 (12.71)	1649 (93.64)	5387 (97.15)	2614 (82.62)	<0.001
**Medication, n (%)**					
Angiotensin-converting-enzyme inhibitors	439 (10.6)	296 (16.8)	663 (12.0)	710 (22.4)	<0.001
Angiotensin II receptor blockers	1123 (27.1)	455 (25.8)	513 (9.3)	544 (17.2)	<0.001
Diuretics	957 (23.1)	751 (42.7)	1532 (27.6)	2118 (66.9)	<0.001
Oral hypoglycemic agents	997 (24.1)	455 (25.8)	527 (9.5)	596 (18.8)	<0.001
Insulin	550 (13.3)	490 (27.8)	983 (17.7)	1126 (35.6)	<0.001
NSAIDs	1039 (25.1)	680 (38.6)	2514 (45.3)	1519 (48.0)	<0.001
Radiocontrast	979 (23.6)	412 (23.4)	1911 (34.5)	1468 (46.4)	<0.001
**Lab data, median (IQR)**					
Baseline BUN, mg/dL	14.0 (11.0, 23.0)	19.0 (12.0, 42.0)	15.0 (10.5, 29.0)	22.0 (12.7, 44.0)	<0.001
Second BUN, mg/dL	14.0 (11.0, 24.0)	21.5 (13.0, 48.0)	14.0 (10.0, 27.0)	23.0 (13.0, 45.0)	<0.001
Baseline serum creatinine, mg/dL	0.97 (0.76, 1.32)	1.15 (0.83, 2.19)	1.01 (0.78, 1.65)	1.16 (0.80, 2.01)	<0.001
Second serum creatinine, mg/dL	0.96 (0.76, 1.33)	1.15 (0.83, 2.20)	0.99 (0.75, 1.58)	1.20 (0.82, 2.10)	<0.001
Baseline eGFR, ml/min/1.73m^2^	81.7 (51.0, 99.4)	60.1 (25.7, 90.8)	75.9 (38.9, 100.8)	58.3 (29.5, 92.1)	<0.001
Serum albumin, g/dL	4.30 (3.80, 4.60)	3.60 (3.00, 4.05)	3.10 (2.50, 3.60)	2.80 (2.30, 3.30)	<0.001
Hemoglobin, g/dL	13.50 (11.70, 14.90)	12.20 (9.90, 14.00)	12.50 (10.75, 14.15)	10.70 (9.35, 12.43)	<0.001
Sodium, mEq/L	138.0 (137.0, 140.0)	136.0 (133.0, 139.0)	138.0 (135.0, 140.0)	138.0 (134.5, 141.5)	<0.001
Potassium, mEq/L	4.10 (3.80, 4.50)	3.90 (3.60, 4.40)	3.70 (3.40, 4.05)	3.80 (3.35, 4.25)	<0.001
White blood cell count, 10^3^/μL	6.46 (5.24, 7.95)	8.19 (6.13, 10.90)	11.25 (8.27, 14.88)	10.31 (7.43, 14.58)	<0.001
C-reactive protein, mg/dL	0.17 (0.06, 0.70)	0.97 (0.22, 4.85)	1.60 (0.29, 7.15)	4.51 (1.17, 12.26)	<0.001
**Summary measures of baseline and second S-Cre**					
Time interval (hours)	0.00 (0.00, 0.43)	4.63 (2.57, 8.05)	8.53 (6.15, 12.13)	8.68 (5.36, 12.85)	<0.001
Difference (ΔS-Cre)	0.04 (0.02, 0.07)	0.10 (0.04, 0.15)	0.13 (0.07, 0.26)	0.10 (0.06, 0.21)	<0.001
Percent change (ΔS-Cre%)	3.66 (1.79, 6.82)	6.00 (2.74, 11.11)	12.24 (6.20, 21.01)	9.09 (4.26, 17.12)	<0.001
**Outcome**					
Deaths before 2017–12–31	736 (17.8)	677 (38.4)	2353 (42.4)	1928 (60.9)	<0.001
3-year all-cause deaths	423 (10.2)	410 (23.3)	1719 (31.0)	1563 (49.4)	<0.001
1-year all-cause deaths	187 (4.5)	244 (13.9)	1219 (22.0)	1211 (38.3)	<0.001
30-day all-cause deaths	14 (0.3)	41 (2.3)	604 (10.9)	634 (20.0)	<0.001

^a^*p*-values are calculated by Kruskal-Wallis test for continuous variables and chi-square test for categorical variables.

Abbreviations: BUN, blood urea nitrogen**;** eGFR, estimated glomerular filtration rate; ICD, Internal Classification of Disease; NSAID, Nonsteroidal anti-inflammatory drugs.

**Figure 2:**
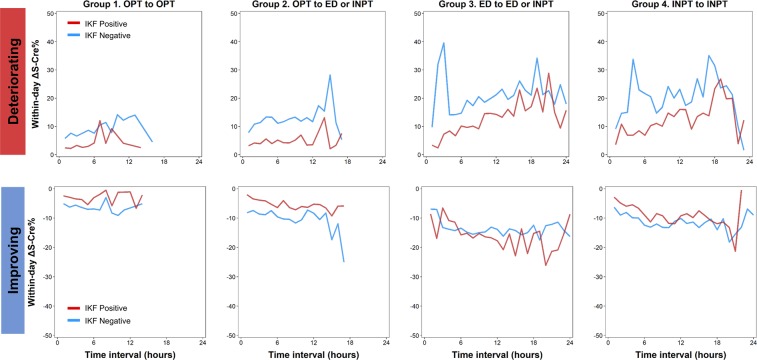
Percent change in S-Cre levels repeated within 24 hours (within-day ΔS-Cre%) over sampling time interval by patients’ service transition patterns. Red line: baseline IKF positive; blue line: baseline IKF negative. IKF, impaired kidney function; S-Cre, serum creatinine.

**Figure 3:**
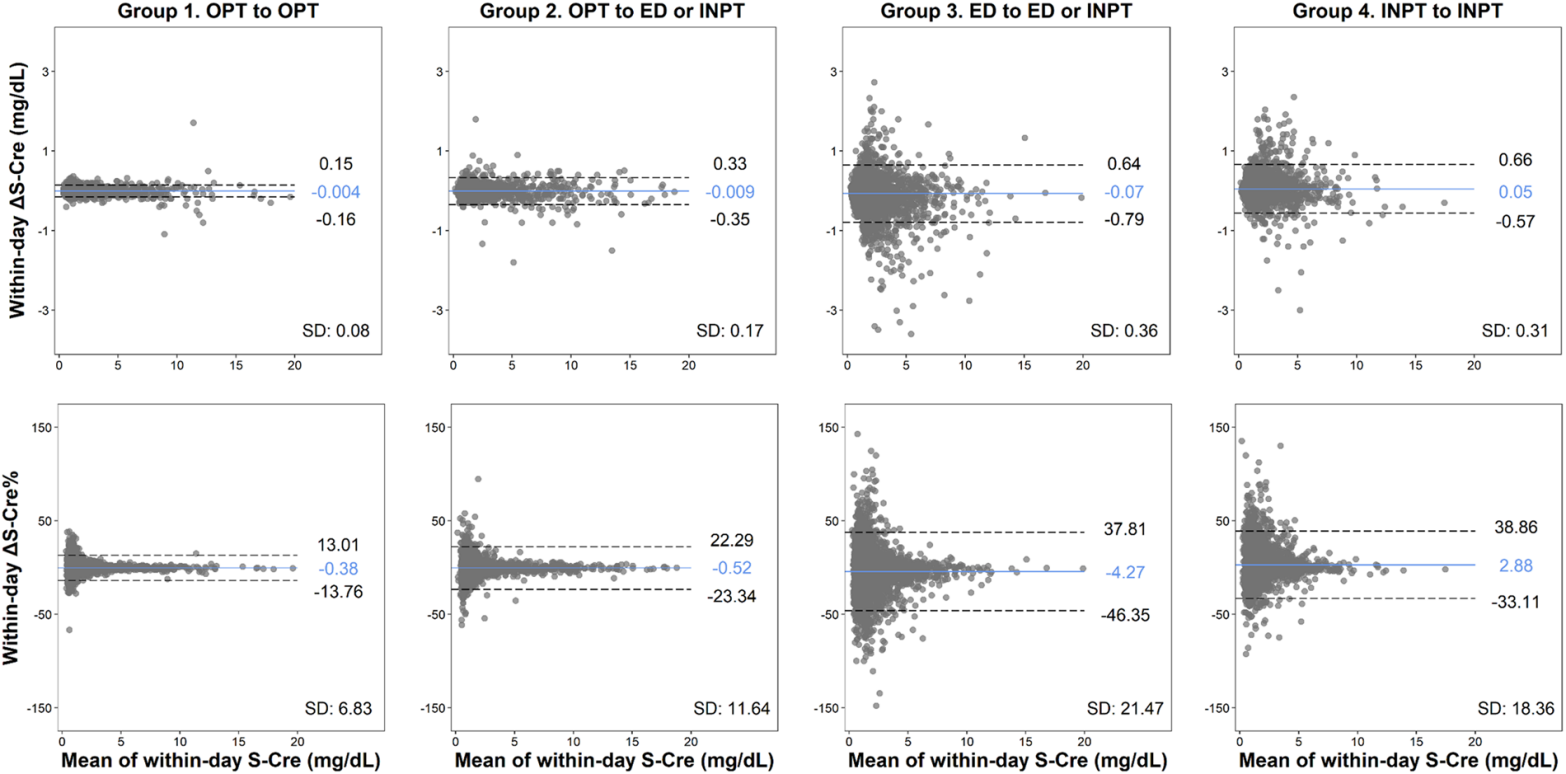
Bland-Altman plots of the difference and percent change between the baseline and the second S-Cre level by patient’s service transition patterns. ED, emergency department; INPT, inpatient; OPT, outpatient; S-Cre, serum creatinine.

The mean time intervals between the first and second S-Cre measurements were: 0 h, Group 1; 4.63 h, Group 2; 8.53 h, Group 3; and 8.68 h, Group 4. When stratifying by service group and IKF status, the magnitude of ΔS-Cre% and ΔS-Cre increased as the time interval increased, particularly in patients from Groups 3 and 4 who had decreasing change in S-Cre levels (Fig. 2 and Supplementary Fig. 1). The magnitude of within-day ΔS-Cre% was greater among patients without IKF and within-day ΔS-Cre was greater among patients with IKF (Fig. 2 and Supplementary Fig. 1). The spikes in Fig. 2 and Supplementary Fig. 1 represent the most prominent variation in kidney function occurring at a specific time interval. Generally, spikes in ΔS-Cre% occurred early in Groups 3 and 4 but late in Group 2. Spikes were less frequent among patients with improving ΔS-Cre% (Fig. 2). However, the spikes in ΔS-Cre were more likely to occur late (Supplementary Fig. 1). When using the Bland–Altman plot to analyze the concordance of the two S-Cre values by service transition groups, the agreement intervals became wider, from SD 6.83% (Group 1) and 11.64% (Group 2) to 21.47% (Group 3) and 18.36% (Group 4). Additionally, there was a trend for the second S-Cre values to improve when the means of the two S-Cre values increased (Fig. 3).

In the overall population, the adjusted hazard ratio (aHR) for 30-day all-cause mortality for each 5% change (within-day ΔS-Cre%) was 1.06 (95% CI: 1.04, 1.07) and 0.1 mg/dL change (within-day ΔS-Cre) was 1.06 (95% CI: 1.04, 1.08) (Table 2 and Supplementary Table 3). The corresponding aHR for 3-year all-cause mortality was 1.02 (95% CI: 1.01, 1.03) and 1.00 (95% CI: 0.99, 1.01) (Supplementary Table 4 and Supplementary Table 5). Among patients with deteriorating ΔS-Cre%, the aHR of per 5% change in ΔS-Cre% for 30-day all-cause mortality was 1.08 (95% CI: 1.06, 1.10) and 3-year all-cause mortality was 1.03 (95% CI: 1.02, 1.04) (Table 2 and Supplementary Table 3). In Group 3, the effect sizes increased to 1.11 (95% CI: 1.07, 1.15) for 30-day all-cause mortality and 1.03 (95% CI: 1.02, 1.05) for 3-year all-cause mortality (Table 2 and Supplementary Table 3). The significant risk pattern was only observed among patients of service Groups 3 and 4. Moreover, improving ΔS-Cre% was associated with lower risk of 3-year all-cause mortality in Group 3 only (Supplementary Table 4). In Group 1, ΔS-Cre% and mortality was not associated. After further adjusted for time intervals between first and second S-Cre measurements, the main inferences remained robust (Supplementary Table 6 and Supplementary Table 7).

**Table 2:** Hazard ratios (95% confidence interval) of 1-year (for Groups 1 and 2) and 30-day (for Groups 3 and 4) all-cause mortality according to every 5% change in S-Cre levels repeated within 24 hours. ED, emergency department; INPT, inpatient; OPT, outpatient; S-Cre, serum creatinine.

	Case/N	Mortality (%)	Crude HR (95% CI)	Model 1		Model 2		Model 3	
				Adjusted HR (95% CI)	*p*-value	Adjusted HR (95% CI)	*p* -value	Adjusted HR (95% CI)	*p* -value
**1-year mortality**									
Overall	2894/14912	19.4%	1.04 (1.03, 1.04)	1.05 (1.05, 1.06)	<0.001	1.04 (1.03, 1.05)	<0.001	1.03 (1.02, 1.04)	<0.001
Deteriorating	1459/6753	21.6%	1.04 (1.03, 1.05)	1.06 (1.05, 1.07)	<0.001	1.05 (1.04, 1.05)	<0.001	1.03 (1.03, 1.04)	<0.001
Improving	1435/8159	17.6%	1.07 (1.04, 1.09)	1.03 (1.01, 1.06)	0.011	0.98 (0.95, 1.01)	0.167	0.97 (0.94, 1.00)	0.041
**Group 1 (OPT to OPT)**									
Overall	187/4145	4.5%	0.95 (0.80, 1.13)	1.06 (0.89, 1.26)	0.537	1.03 (0.86, 1.24)	0.743	0.97 (0.81, 1.17)	0.768
Deteriorating	95/1882	5.0%	0.98 (0.80, 1.20)	1.06 (0.85, 1.31)	0.616	1.01 (0.80, 1.28)	0.916	0.95 (0.75, 1.20)	0.639
Improving	92/2263	4.1%	0.89 (0.66, 1.21)	1.02 (0.73, 1.42)	0.916	1.01 (0.73, 1.41)	0.936	0.98 (0.69, 1.40)	0.917
**Group 2 (OPT to ED or INPT)**									
Overall	244/1761	13.9%	0.99 (0.92, 1.07)	1.05 (0.99, 1.12)	0.099	1.06 (1.00, 1.13)	0.063	1.05 (0.98, 1.12)	0.164
Deteriorating	102/782	13.0%	1.01 (0.93, 1.10)	1.06 (0.98, 1.15)	0.117	1.07 (0.99, 1.16)	0.076	1.06 (0.97, 1.15)	0.221
Improving	142/979	14.5%	0.92 (0.79, 1.08)	1.05 (0.89, 1.23)	0.559	1.01 (0.86, 1.18)	0.929	1.01 (0.86, 1.20)	0.882
**30-day mortality**									
Overall	1304/14912	8.7%	1.09 (1.07, 1.10)	1.10 (1.08, 1.11)	<0.001	1.07 (1.05, 1.08)	<0.001	1.06 (1.04, 1.07)	<0.001
Deteriorating	745/6753	11.0%	1.10 (1.08, 1.12)	1.12 (1.10, 1.15)	<0.001	1.09 (1.07, 1.11)	<0.001	1.08 (1.06, 1.10)	<0.001
Improving	559/8159	6.9%	1.11 (1.06, 1.16)	1.07 (1.03, 1.12)	0.001	0.99 (0.95, 1.04)	0.805	0.99 (0.94, 1.04)	0.633
**Group 3 (ED to ED or INPT)**									
Overall	604/5545	10.9%	1.05 (1.03, 1.08)	1.06 (1.04, 1.08)	<0.001	1.06 (1.04, 1.09)	<0.001	1.06 (1.04, 1.09)	<0.001
Deteriorating	270/2184	12.4%	1.08 (1.04, 1.11)	1.09 (1.06, 1.13)	<0.001	1.10 (1.06, 1.15)	<0.001	1.11 (1.07, 1.15)	<0.001
Improving	334/3361	9.9%	0.99 (0.93, 1.05)	0.97 (0.91, 1.03)	0.362	0.96 (0.9, 1.03)	0.262	0.96 (0.90, 1.03)	0.261
**Group 4 (INPT to INPT)**									
Overall	634/3164	20.0%	1.02 (1.00, 1.04)	1.04 (1.02, 1.06)	<0.001	1.04 (1.02, 1.06)	<0.001	1.03 (1.01, 1.06)	0.002
Deteriorating	437/1736	25.2%	1.03 (1.01, 1.06)	1.05 (1.02, 1.08)	<0.001	1.05 (1.02, 1.08)	<0.001	1.06 (1.03, 1.09)	<0.001
Improving	197/1428	13.8%	0.93 (0.82, 1.05)	0.95 (0.83, 1.08)	0.434	0.90 (0.78, 1.04)	0.150	0.95 (0.81, 1.10)	0.481

Model 1: Adjusted for gender, body mass index, diabetes, hypertension, impaired kidney function, noncancerous catastrophic illness, acute kidney failure, baseline eGFR.

Model 2: Further adjusted for medications listed in Table 1 including fluid therapy between two S-Cre measurements.

Model 3: Further adjusted for baseline blood urea nitrogen, C-reactive protein, white blood cell count, serum albumin, hemoglobin.

**Figure 4:**
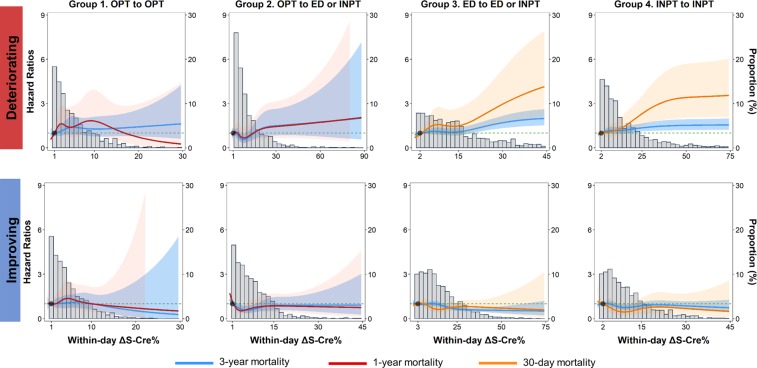
Adjusted hazard ratios (aHRs) for 30-day (red line), 1-year (dark-red line), and 3-year (blue line) all-cause mortality according to the percent change in S-Cre levels repeated within 24 hours (within-day ΔS-Cre%) by patients’ service transition patterns and variation directions (deteriorating vs. improving). Solid lines represent aHRs based on restricted cubic splines for within-day ΔS-Cre%, with knots at the 5^th^, 25^th^,, 50th, 75^th^, and 95^th^ percentiles. Shaded areas represent the upper and lower 95% confidence intervals. Reference was set at 10th percentile of ΔS-Cre% levels. Variables adjusted are the same as that shown in Model 3 of Table 2. S-Cre, serum creatinine.

In the dose-response analysis, positive relationships were observed between deteriorating ΔS-Cre and ΔS-Cre% and 30-day and 3-year mortality in Group 3 and 4 patients (Fig. 4 and Supplementary Fig. 2, upper panel). Negative relationships were observed between improving ΔS-Cre and ΔS-Cre% and 30-day and 3-year mortality in Group 3 and 4 patients (Fig. 4 and Supplementary Fig. 2, lower panel). By contrast, the magnitude of improving ΔS-Cre and ΔS-Cre% was not associated with short- or long-term mortality in Group 1 patients (Fig. 4 and Supplementary Fig. 2, lower panel). In Group 3 and 4 patients, the optimal cut-offs for the prediction of 30-day and 3-year mortality were determined to be approximately 0.2 mg/dL increase for ΔS-Cre and 20% increase for ΔS-Cre% (Supplementary Figs. 3 and 4, upper panel). In patients with IKF, the corresponding cut-off for deteriorating ΔS-Cre% dropped to 10–13%; however, among Group 4 patients with IKF, the clinical significance threshold of ΔS-Cre remained consistent at 0.22 (Supplementary Table 8).

## Discussion

This real-world study provides a thorough understanding of the clinical significance of 24-hour ΔS-Cre and ΔS-Cre%, which can be used to inform diagnostic criteria of both outpatient- and inpatient-AKI (AKI_OPT_ and AKI_INPT_). The clinical significance of within-day ΔS-Cre and ΔS-Cre% is different in inpatient and outpatient settings; the positive linear relationship between all-cause mortality and deteriorating ΔS-Cre or ΔS-Cre% is only observed in the inpatient settings, regardless of whether the all-cause-mortality is short- or long-term or the change in S-Cre is very small. In addition, a 0.2 mg/dL (ΔS-Cre) or 20% (ΔS-Cre%) change is clinically meaningful in predicting the risk of all-cause mortality in inpatient settings. However, for patients with IKF, the clinical threshold of ΔS-Cre% should be reduced to approximately 10%. The physiological daily variation in S-Cre is rarely greater than 10% and the variation can be up to 30% without any prognostic significance. Nevertheless, a 30% change in S-Cre within 24 hours should raise clinical vigilance in the outpatient setting.

Existing evidence separates the variability of S-Cre into within-person, between-person, and analytical variations (CV_A_). Reinhard *et al*. found that the analytical variation of S-Cre was stable at less than 2% across a wide range of kidney functions; however, the within-person biological variation (CV_I_) could vary from 4.7% to 8.9% for individuals with and without impaired renal function^9^. Similar observations were also made by Carter *et al*., who found a low CV_A_ of 0.6% and a CV_I_ from 5.1% to above 6% in patients with impaired kidney function (eGFR<60 mL/min/1.73 m^2^) and proteinuria^10^. Moreover, previous studies suggested that a low index of individuality (II) for S-Cre supports stable S-Cre levels within an individual over time^10,11^. However, few studies have associated S-Cre variability, particularly within 24 or 48 hours, with mortality using real-world data. Therefore, the current diagnostic criteria for AKI_INPT_ are empirical and conservatively sensitivity-centered^12^. Two articles have shown that small differences of 0.3–0.4 mg/dL in S-Cre are associated with in-hospital mortality and 30-day post-surgical mortality; however, neither of them evaluated the effects of within-day S-Cre variation^13,14^.

The clinical significance of changes in within-day S-Cre variation based on different clinical settings reflects hidden mechanisms underlying the observed inferences. First, in the outpatient setting, a change of up to 30% in S-Cre carries no mortality risk. By contrast, the same magnitude of change in S-Cre is associated with significantly higher risk of death among inpatients. This difference can be explained by the fluid optimization that occurs during the interval between S-Cre sampling in the inpatient setting, which is unusual among outpatients. Second, in the ED setting (Group 3), we found that increasing ΔS-Cre% in improving direction was uniquely associated with protective effects. Whether such acute recovery represents the protective effects of early intervention or the individual’s rapid and efficient compensatory response requires further research. Third, blood samples from patients who had their S-Cre measured twice within 24 hours in the outpatient setting (Group 1) are perfect for quantifying real-world CV_A_, particularly in paired samples with an examination time difference of less than one hour. In Group 1, the median ΔS-Cre and ΔS-Cre% were 0.04 and 3.41%, respectively, and the 99-percentile of ΔS-Cre and ΔS-Cre% was 0.23 mg/dL and 21.7%, respectively, providing insights into the maximal extent of the physiological variation in S-Cre.

Our results facilitate the in-time diagnosis of AKI by defining actionable cut-offs that accurately differentiate physiological variations from pathological variations of within-day S-Cre. Our findings support the idea that variation in CV_I_ represents the function of biological variation, and an individual’s compensatory capacity to maintain kidney creatinine clearance in common etiologies of AKI associated with renal hypoperfusion or ischemia, such as dehydration, blood loss, or cardiogenic shock. In patients with sepsis, AKI may occur from concomitant systemic hypoperfusion and intrarenal vasodilatation, resulting in acute eGFR change. In the above-mentioned clinical conditions, intravenous hydration and hemodynamic supportive measures remain the treatment of choice^15,16^. Patients in Groups 3 (ED-to-INPT) and 4 (INPT-to-INPT) are likely to have received fluid replacement between the within-day measurements of S-Cre. Consequently, S-Cre should theoretically decrease in response to the hemodynamic load and dilution effects due to fluid expansion^17^. In Group 3 and 4, deteriorating rather than improving ΔS-Cre or ΔS-Cre% within 24 hours suggests decompensating renal autoregulation to counterbalance the effects of systemic derangement and predisposes individuals to poor clinical outcomes. By contrast, among patients who had their first S-Cre measured at the ED (Group 3), improving ΔS-Cre or ΔS-Cre% suggests the individual’s preserved kidney compensatory capacity to respond to the initial resuscitation and, therefore, is associated with favorable outcomes. On the other hand, in the ambulatory population, the CV_I_ of S-Cre within 24 hours is small and reflects mainly physiological fluctuation; therefore, in the outpatient setting, a higher cut-off, for example, 30% (99 percentile level plus 10%) (Fig. 1), to alarm pathologic variation is rational in patients with normal kidney function and happened to be consistent with findings reported in 1971^4^. A lower within-day ΔS-Cre% cut-off, for example, 20%, in inpatients who have been admitted or have received empirical hydration is appropriate to identify patients with clinically meaningful kidney decompensation. The within-day ΔS-Cre% cut-off values of 30% and 20% for outpatients and inpatients, respectively, provide the first evidence-based threshold for the identification of clinically significant kidney decompensation. These values imply that for AKI_INPT_, the existing empirical diagnostic criteria of ΔS-Cre > 0.3 mg/dL within 48 hours or ΔS-Cre% > 50% over a 7-day period may be relatively late for AKI_INPT_.

This study had several limitations. First,the residual confounding factors could not be completely excluded, particularly, the reasons for the patients receiving two S-Cre measurements on the same day. However, by stratifying the study population according to different service utilization patterns, this bias could be better controlled and understood. This approach further generates novel insights into the clinical meaning of within-day ΔS-Cre or ΔS-Cre% based on different clinical settings. Second, S-Cre in this study was measured by Jaffe method rather than the IDMS (isotope dilution-mass spectrometry) traceable enzymatic method, which may overestimate S-Cre, resulting in misclassification of IKF. Other limitations include the over-adjustment for variables that could be in the causal pathway and the possibility that these results should not be generalized to other ethnic populations.

In conclusion, increasing within-day ΔS-Cre or ΔS-Cre% above 0.2 mg/dL or 20%, respectively, were associated with increased all-cause mortality, particularly in inpatient settings. In outpatient settings, increasing within-day ΔS-Cre or ΔS-Cre% above 0.3 mg/dL or 30%, respectively, should raise concerns and trigger vigilance monitoring of kidney function. Moreover, it is the time to reconsider using clinical settings to classify AKI (eg AKI_INPT_ and AKI_OPT_) rather than location (community acquired vs. hospital acquired).

## Methods

### Study population

In 2017, China Medical University Hospital (CMUH) established the Clinical Research Data Repository (CRDR) to verify and validate data from a variety of clinical sources to unify the trackable patient information generated during the healthcare process. Between January 1, 2003 and December 31, 2016, CMUH-CRDR had accumulated single unified records of 2 66 0472 patients who had sought medical care at CMUH. The data collected in the CMUH-CRDR includes administrative and demographic information, diagnoses, medical and surgical procedures, prescription drugs, laboratory measurements, physiological data, and status of catastrophic illnesses^18^. The interoperability of the CMUH-CRDR has expanded access to national population-based health-related databases (e.g., mortality database). The present cohort was composed of patients aged 18–90 years who had ever received two S-Cre measurements on the same day at two different analytical time points. The date of repeated S-Cre measurement was the index date. If patients had multiple events of within-day repeated S-Cre measurements in the CMUH-CRDR, the date of the first event was defined as the index date. We excluded patients who had a history of cancer and those who had undergone dialysis therapy, kidney transplantation, or cardiopulmonary resuscitation within 7 days prior to the index day. The present study included a total of 14 912 patients who were followed up to the date of death or were censored at the corresponding 30-day, 1-year, or 3-year time point after the index date. (Supplementary Fig. 5). The study was approved by the Big Data Center of China Medical University Hospital and the Research Ethical Committee/Institutional Review Board of China Medical University Hospital (CMUH105-REC3–068) and the need to obtain informed consent for the present study was waived by the Research Ethical Committee of China Medical University Hospital.

### Quantification of serum creatinine and its within-day variation

S-Cre levels were measured using the Jaffe rate method at CMUH Central Laboratory using a Beckman UniCel DxC 800 immunoassay system (Beckman Coulter Inc., Brea, CA, USA). The estimated glomerular filtration rate (eGFR) was calculated using the Chronic Kidney Disease-Epidemiology Collaboration equation^19^. Among patients with multiple S-Cre measurements (>2 times) on the same day, we selected the first two S-Cre measurements for final analysis. The within-day S-Cre difference (ΔS-Cre) was calculated by subtracting the first S-Cre value from the second value. The within-day percentage change (ΔS-Cre%) was calculated by dividing the within-day difference by the first S-Cre value and multiplying by 100. IKF was defined by first S-Cre value greater than 1.5 mg/dL for men or 1.3 mg/dL for women.

### Other variables

Sociodemographic variables were retrieved from the CMUH-CRDR. Body mass index (BMI) was calculated using the standard formula: body weight (kg) divided by height squared (m^2^). Diabetes mellitus and hypertension were defined using physicians’ clinical diagnoses, according to the patients’ ICD-9-CM codes and the use of glucose-lowering or anti-hypertensive agents. Baseline comorbidities and medication use, and any relevant biochemical measures, were determined based on information obtained from the CMUH-CRDR respectively within a 1-year and 3-month window prior to case enrollment. Catastrophic illness status was defined as having a condition classified as catastrophic illness by the Ministry of Health and Welfare in Taiwan. The catastrophic illnesses include 30 disease categories such as: malignancy, hemophilia, inherited hemolytic anemias, chronic kidney disease, systemic autoimmune disease, chronic psychiatric disorders, inherited metabolic disorders, congenital anomalies, severe burn, transplantation, multiple sclerosis, liver cirrhosis with complications, rare diseases, and other conditions that require long-term and systemic medical care (https://ws.nhi.gov.tw/001/Upload/293/RelFile/Ebook/English.pdf). Noncancerous catastrophic status was defined by excluding patients who ever had a diagnosis of malignancy verified by National Catastrophic Illness Registry.

### Statistical analyses

The study population was separated into improving (ΔS-Cre < 0) and deteriorating (ΔS-Cre > 0) groups and then assigned into quartile groups based on the quartile cut-offs of the ΔS-Cre or ΔS-Cre%. They were also divided into four groups according to the hospital service where the first and second S-Cre were measured: Group 1, Outpatient (OPT)-to-OPT; Group 2, OPT-to-ED (emergency department) or inpatient (INPT); Group 3, ED-to-ED or INPT; and Group 4, INPT–to-INPT. In the univariable analyses, we separately compared clinical characteristics across quartiles groups of ΔS-Cre% or ΔS-Cre and across four service transition patterns (Group 1–4). Continuous variables were expressed as the median and interquartile range (IQR) and compared using the nonparametric Kruskal–Wallis test due to several biochemical parameters were not normally distributed such as blood urea nitrogen and serum creatinine. Categorical variables were expressed as frequency (percentage) and compared using the chi-square test. The associations between within-day variation (in continuous and quartile categories) and risks of 30-day (Groups 3 and 4), 1-year (Groups 1 and 2), and 3-year (all groups) all-cause mortality were estimated using multivariable Cox regression analysis. The distribution of within-day ΔS-Cre was described according to time and baseline IKF status. Then Bland–Altman analysis was applied to evaluate the agreement between the two S-Cre measurements. To minimize the negative effects of missing data on risk association analysis (e.g., reduced statistical power), we performed multiple imputation through the fully conditional specification method in SAS, an iterative Markov chain Monte Carlo procedure, to replace the missing values for variables in our proposed formula with imputed values. We specified the imputation number as 20 and iteration number as 100^20^. Multivariable Cox regression models, using age as the time scale, were initially adjusted for sociodemographic variables including age, sex, comorbidities, and baseline eGFR, followed by adjusting for baseline medications, and then by adjusting for baseline nutritional and inflammatory markers. The dose–response relationships were further characterized between all-cause mortality and within-day ΔS-Cre variation using a restricted cubic spline model with five knots located at the 5^th^, 25^th^, 50^th^, 75^th^, and 95^th^ percentiles of the ΔS-Cre variation distribution of the four transition groups^21^. To predict 30-day, 1-year, and 3-year mortality, optimal cut-off values were determined for ΔS-Cre and ΔS-Cre% in each service group (Groups 1–4) when the log-rank test statistics was maximal. All statistical analyses were performed in SAS version 9.4 (SAS Institute Inc., Cary, NC, USA) and R version 3.2.3 (R Foundation for Statistical Computing, Vienna, Austria). The 2-sided statistical significance level was set at α = 0.05.

### Ethical approval

The study was approved by the Research Ethical Committee/Institutional Review Board of China Medical University Hospital (CMUH105-REC3-068).

## Supplementary information

Supplementary information.

## Data Availability

The data that support the findings of this study are available on request from the corresponding author, CCK. The data are not publicly available due to them containing information that could compromise research participant privacy.
