# Supplementary material for: 24-hour Serum Creatinine Variation Associates with Short- and Long-Term All-Cause Mortality: A Real-World Insight into Early Detection of Acute Kidney Injury
Source: Sci Rep. 2020 Apr 16;10:6552. doi: 10.1038/s41598-020-63315-x (PMC7162857; doi:10.1038/s41598-020-63315-x)
Supplement: Supplementary file 1 — Supplementary information. [file 41598_2020_63315_MOESM1_ESM.docx]

**Online-Only Supplementary Material**

**Title:** 24-hour Serum Creatinine Variation Associates with Short- and Long-Term All-Cause Mortality: A Real-World Insight into Early Detection of Acute Kidney Injury

**Authors:** Hung-Chieh Yeh; Yen-Chun Lo; I-Wen Ting; Pei-Lun Chu; Shih-Ni Chang; Hsiu-Yin Chiang; Chin-Chi Kuo

**Supplementary Tables**

**Supplementary Table 1a.** Baseline demographic and clinical characteristics based on quartiles of within-day serum creatinine percent change (ΔS-Cre%) among patients with deterioration kidney function (ΔS-Cre% >0).

**Supplementary Table 1b.** Baseline demographic and clinical characteristics based on quartiles of within-day serum creatinine percent change (ΔS-Cre%) among patients with improving kidney function (ΔS-Cre% <0).

**Supplementary Table 2a**. Baseline demographic and clinical characteristics based on quartiles of within-day serum creatinine difference (ΔS-Cre) among patients with deterioration kidney function (ΔS-Cre >0).

**Supplementary Table 2b.** Baseline demographic and clinical characteristics based on quartiles of within-day serum creatinine difference (ΔS-Cre) among patients with improving kidney function (ΔS-Cre <0).

**Supplementary Table 3.** Hazard ratios (95% confidence interval) of 3-year all-cause mortality according to every 5% change in S-Cre levels repeated within 24 hours. ED, emergency department; INPT, inpatient; OPT, outpatient; S-Cre, serum creatinine.

**Supplementary Table 4.** Hazard ratios (95% confidence interval) of 1-year (for Groups 1 and 2) and 30-day (for Groups 3 and 4) all-cause mortality according to every 0.1 mg/dL change in S-Cre levels repeated within 24 hours. ED, emergency department; INPT, inpatient; OPT, outpatient; S-Cre, serum creatinine.

**Supplementary Table 5.** Hazard ratios (95% confidence interval) of 3-year all-cause mortality according to every 0.1 mg/dL change in S-Cre levels repeated within 24 hours. ED, emergency department; INPT, inpatient; OPT, outpatient; S-Cre, serum creatinine.

**Supplementary Table 6.** Sensitivity analysis of 3-year all-cause mortality according to every 5% change in S-Cre levels repeated within 24 hours after additionally adjusted for time intervals. ED, emergency department; INPT, inpatient; OPT, outpatient; S-Cre, serum creatinine.

**Supplementary Table 7.** Sensitivity analysis of 3-year all-cause mortality according to every 0.1 mg/dL change in S-Cre levels repeated within 24 hours after additionally adjusted for time intervals. ED, emergency department; INPT, inpatient; OPT, outpatient; S-Cre, serum creatinine.

**Supplementary Table 8.** Summary of cut-off determination of ΔS-Cre% and ΔS-Cre for 30-day, 1-year, and 3-year all-cause mortality by patients’ service transition patterns and baseline status of impaired kidney function (IKF). Numbers in bold represent significant effects of ΔS-Cre% or ΔS-Cre levels on all-cause mortality. ΔS-Cre, difference between baseline and second serum creatinine; ΔS-Cre%, percent change between baseline and second serum creatinine.

**Supplementary Figures**

**Supplementary Figure 1.** Difference in S-Cre levels repeated within 24 hours (within-day ΔS-Cre) over sampling time interval by patients’ service transition patterns. Red line: baseline IKF positive; blue line: baseline IKF negative. IKF, impaired kidney function; S-Cre, serum creatinine.

**Supplementary Figure 2.** Adjusted hazard ratios (aHRs) for 30-day (red line), 1-year (dark-red line), and 3-year (blue line) all-cause mortality according to the difference in S-Cre levels repeated within 24 hours (within-day ΔS-Cre) by patients’ service transition patterns and variation directions (deteriorating vs. improving). Solid lines represent aHRs based on restricted cubic splines for within-day ΔS-Cre, with knots at the 5^th^, 25^th^, 50^th^, 75^th^, and 95^th^ percentiles. Shaded areas represent the upper and lower 95% confidence intervals. Reference was set at 10th percentile of ΔS-Cre levels. Variables adjusted are the same as that shown in Model 3 of Table 3. S-Cre, serum creatinine.

**Supplementary Figure 3.** Cut-off determination of ΔS-Cre% for 30-day and 1-year all-cause mortality by patients’ service transition patterns**.** Dots in black and gray represent significant and nonsignificant effects of ΔS-Cre% levels on all-cause mortality, respectively, shown in Model 3 of Table 3. ΔS-Cre%, percent change between baseline and second serum creatinine.

**Supplementary Figure 4.** Cut-off determination of ΔS-Cre for 30-day and 1-year all-cause mortality by patients’ service transition patterns**.** Dots in black and gray represent significant and nonsignificant effects of ΔS-Cre levels on all-cause mortality, respectively, shown in Model 3 of Table 3. ΔS-Cre, difference between baseline and second serum creatinine.

**Supplementary Figure 5.** Study flow chart of 14912 patients who were included in analysis. ESRD, end stage renal disease; PD, peritoneal dialysis; HD, hemodialysis; CVVH, continuous venovenous hemofiltration; DFPP, double filtration plasmapheresis; APD, automated peritoneal dialysis; CPCR, cardiopulmonary-cerebral resuscitation; S-Cre, serum creatinine; OPT, outpatient; ED, emergency department; INPT, inpatient.

**Supplementary Table 1a.** Baseline demographic and clinical characteristics based on quartiles of within-day serum creatinine percentage change (ΔS-Cre%) among patients with deterioration kidney function (ΔS-Cre% >0).

| **Variables** |  |  | **1st Quartile** | **2nd Quartile** | **3rd Quartile** | **4th Quartile** |  |  |
| --- | --- | --- | --- | --- | --- | --- | --- | --- |
| **\| ΔS-Cre% \|** | **N** | **Total** | **<3.25%** | **3.25%≤~<7.69%** | **7.69%≤~<15.78%** | ≥**15.78%** | ***p*-value^a^** | ***p*-value for trend^b^** |
| **Sample size** |  | **6753** | **1689** | **1682** | **1693** | **1689** |  |  |
| **Demographics, median (IQR)** |  |  |  |  |  |  |  |  |
| Age, years | 6753 | 60.4 (46.6, 74.2) | 63.4 (51.0, 75.8) | 60.7 (48.0, 74.0) | 58.3 (43.2, 72.8) | 58.3 (43.9, 73.6) | <0.001 | <0.001 |
| Men, n (%) | 6753 | 3973 (58.83) | 1038 (61.46) | 986 (58.62) | 964 (56.94) | 985 (58.32) | 0.038 | 0.038 |
| Body mass index (kg/m^2^) | 2831 | 24.2 (21.3, 27.2) | 24.3 (21.4, 27.4) | 24.4 (21.6, 27.3) | 24.0 (21.3, 27.0) | 23.9 (21.0, 26.8) | 0.141 | <0.001 |
| **Hospital service use at second S-Cre measurement, n (%)** | 6753 |  |  |  |  |  | <0.001 | − |
| Outpatient |  | 2015 (29.84) | 862 (51.04) | 631 (37.51) | 407 (24.04) | 115 (6.81) |  |  |
| Emergency department |  | 1527 (22.61) | 338 (20.01) | 344 (20.45) | 415 (24.51) | 430 (25.46) |  |  |
| Inpatient |  | 3211 (47.55) | 489 (28.95) | 707 (42.03) | 871 (51.45) | 1144 (67.73) |  |  |
| General ward |  | 1342 (41.79) | 224 (45.81) | 300 (42.43) | 366 (42.02) | 452 (39.51) |  |  |
| Intensive care unit |  | 1869 (58.21) | 265 (54.19) | 407 (57.57) | 505 (57.98) | 692 (60.49) |  |  |
| Fluid therapy between two measurements | 6753 | 3580 (53.01) | 541 (32.03) | 763 (45.36) | 995 (58.77) | 1281 (75.84) | <0.001 | <0.001 |
| Fluid therapy on the index day | 6753 | 4705 (69.67) | 838 (49.62) | 1071 (63.67) | 1274 (75.25) | 1522 (90.11) | <0.001 | <0.001 |
| **Comorbidities, n (%)** |  |  |  |  |  |  |  |  |
| Impaired kidney function | 6753 | 1829 (27.08) | 723 (42.81) | 512 (30.44) | 310 (18.31) | 284 (16.81) | <0.001 | <0.001 |
| Acute kidney failure (ICD-9 codes 584.5-584.9) | 6753 | 360 (5.33) | 137 (8.11) | 96 (5.71) | 62 (3.66) | 65 (3.85) | <0.001 | <0.001 |
| Diabetes mellitus | 6753 | 1798 (26.63) | 580 (34.34) | 481 (28.60) | 372 (21.97) | 365 (21.61) | <0.001 | <0.001 |
| Hypertension | 6753 | 2061 (30.52) | 702 (41.56) | 552 (32.82) | 415 (24.51) | 392 (23.21) | <0.001 | <0.001 |
| Noncancerous catastrophic illness status | 6753 | 1161 (17.19) | 277 (16.40) | 262 (15.58) | 278 (16.42) | 344 (20.37) | <0.001 | <0.001 |
| **Medication, n (%)** |  |  |  |  |  |  |  |  |
| Angiotensin-converting-enzyme inhibitors | 6753 | 1094 (16.20) | 278 (16.46) | 289 (17.18) | 247 (14.59) | 280 (16.58) | 0.577 | 0.577 |
| Angiotensin II receptor blockers | 6753 | 1317 (19.50) | 486 (28.77) | 368 (21.88) | 249 (14.71) | 214 (12.67) | <0.001 | <0.001 |
| Diuretics | 6753 | 2683 (39.73) | 702 (41.56) | 687 (40.84) | 585 (34.55) | 709 (41.98) | 0.343 | 0.343 |
| Oral hypoglycemic agents | 6753 | 1281 (18.97) | 427 (25.28) | 370 (22.00) | 256 (15.12) | 228 (13.50) | <0.001 | <0.001 |
| Insulin | 6753 | 1510 (22.36) | 429 (25.40) | 371 (22.06) | 315 (18.61) | 395 (23.39) | 0.036 | 0.036 |
| NSAIDs | 6753 | 2923 (43.28) | 573 (33.93) | 660 (39.24) | 789 (46.60) | 901 (53.35) | <0.001 | <0.001 |
| Radiocontrast | 6753 | 2146 (31.78) | 464 (27.47) | 503 (29.90) | 541 (31.96) | 638 (37.77) | 0.002 | 0.002 |
| **Lab data, median (IQR)** |  |  |  |  |  |  |  |  |
| Baseline BUN, mg/dL | 6103 | 16.5 (11.0, 31.0) | 22.0 (13.0, 51.0) | 17.0 (11.0, 35.0) | 15.0 (10.5, 25.0) | 15.0 (11.0, 24.0) | <0.001 | <0.001 |
| Second BUN, mg/dL | 4875 | 17.0 (11.0, 34.0) | 24.0 (13.0, 57.5) | 18.0 (12.0, 38.0) | 15.0 (11.0, 27.0) | 17.0 (11.0, 27.0) | <0.001 | <0.001 |
| Baseline S-Cre, mg/dL | 6753 | 0.96 (0.73, 1.53) | 1.23 (0.86, 2.62) | 1.01 (0.76, 1.69) | 0.90 (0.70, 1.23) | 0.85 (0.63, 1.20) | <0.001 | <0.001 |
| Second S-Cre, mg/dL | 6753 | 1.09 (0.82, 1.76) | 1.26 (0.88, 2.65) | 1.06 (0.81, 1.76) | 1.00 (0.80, 1.37) | 1.11 (0.81, 1.66) | <0.001 | <0.001 |
| Baseline eGFR, ml/min/1.73m^2^ | 6753 | 78.6 (41.2, 100.9) | 55.3 (20.4, 91.6) | 74.2 (37.1, 99.7) | 85.0 (54.7, 103.1) | 87.7 (56.8, 107.3) | <0.001 | <0.001 |
| Serum albumin, g/dL | 3938 | 3.20 (2.60, 3.90) | 3.60 (2.90, 4.20) | 3.40 (2.60, 4.10) | 3.10 (2.50, 3.90) | 3.00 (2.40, 3.55) | <0.001 | <0.001 |
| Hemoglobin, g/dL | 6087 | 12.3 (10.3, 14.0) | 11.8 (9.80, 13.9) | 12.3 (10.2, 14.0) | 12.6 (10.6, 14.2) | 12.2 (10.4, 13.9) | <0.001 | <0.001 |
| Sodium, mEq/L | 5625 | 138 (135, 140) | 138 (134.5, 140) | 138 (135, 140) | 138 (135, 140) | 138 (135, 141) | 0.226 | 0.001 |
| Potassium, mEq/L | 5861 | 3.83 (3.45, 4.30) | 4.05 (3.60, 4.50) | 3.90 (3.50, 4.33) | 3.70 (3.40, 4.10) | 3.75 (3.40, 4.13) | <0.001 | <0.001 |
| White blood cell count, 10^3^/μL | 6000 | 9.23 (6.70, 13.05) | 8.03 (6.14, 11.23) | 8.62 (6.45, 12.28) | 9.62 (6.89, 13.28) | 10.54 (7.72, 14.56) | <0.001 | <0.001 |
| C-reactive protein, mg/dL | 4230 | 1.84 (0.32, 7.09) | 1.16 (0.23, 6.18) | 1.53 (0.30, 6.55) | 1.99 (0.34, 7.29) | 2.27 (0.45, 7.86) | <0.001 | <0.001 |
| **Summary measures of baseline and second S-Cre, median (IQR)** |  |  |  |  |  |  |  |  |
| Time interval (hours) | 6753 | 5.72 (1.27, 9.55) | 1.53 (0.00, 6.41) | 4.21 (0.02, 8.80) | 6.32 (3.00, 10.13) | 7.93 (5.76, 11.27) | <0.001 | <0.001 |
| Difference (ΔS-Cre) | 6753 | 0.10 (0.04, 0.19) | 0.02 (0.01, 0.04) | 0.06 (0.04, 0.10) | 0.10 (0.08, 0.14) | 0.24 (0.16, 0.40) | <0.001 | <0.001 |
| Percent change (ΔS-Cre%) | 6753 | 7.48 (3.30, 14.14) | 1.79 (1.13, 2.56) | 5.13 (4.17, 6.23) | 10.0 (8.77, 11.9) | 20.9 (16.8, 27.7) | <0.001 | <0.001 |
| **Outcome, n (%)** |  |  |  |  |  |  |  |  |
| All-cause deaths before 2017-12-31 | 6753 | 2733 (40.47) | 622 (36.83) | 627 (37.28) | 665 (39.28) | 819 (48.49) | <0.001 | <0.001 |
| 3-year all-cause deaths | 6753 | 2021 (29.93) | 442 (26.17) | 463 (27.53) | 474 (28.00) | 642 (38.01) | <0.001 | <0.001 |
| 1-year all-cause deaths | 6753 | 1459 (21.61) | 282 (16.70) | 329 (19.56) | 346 (20.44) | 502 (29.72) | <0.001 | <0.001 |
| 30-day all-cause deaths | 6753 | 745 (11.03) | 111 (6.57) | 144 (8.56) | 170 (10.04) | 320 (18.95) | <0.001 | <0.001 |

^a^*p*-alues are calculated by Kruskal-wallis test for continuous variables and chi-square test for categorical variables.

^b^*p*-values for trend are calculated by Spearman’s correlation for continuous variables and by Cochran-Armitage trend test for binary variables.

**Abbreviations:** BUN, blood urea nitrogen; eGFR, estimated glomerular filtration rate; ICD, International Classification of Disease; NSAID, Nonsteroidal anti-inflammatory drugs; S-Cre, serum creatinine

**Supplementary Table 1b.** Baseline demographic and clinical characteristics based on quartiles of within-day serum creatinine percent change (ΔS-Cre%) among patients with improving kidney function (ΔS-Cre% <0).

| **Variables** |  |  | **1st Quartile** | **2nd Quartile** | **3rd Quartile** | **4th Quartile** |  |  |
| --- | --- | --- | --- | --- | --- | --- | --- | --- |
| **\| ΔS-Cre% \|** | **N** | **Total** | **<3.30%** | **3.30%≤~<7.48%** | **7.48%≤~<14.14%** | ≥**14.14%** | ***p*-value^a^** | ***p*-value for trend^b^** |
| **Sample size** |  | **8159** | **2040** | **2038** | **2041** | **2040** |  |  |
| **Demographics, median (IQR)** |  |  |  |  |  |  |  |  |
| Age, years | 8159 | 59.0 (44.8, 73.3) | 61.9 (48.8, 73.9) | 61.2 (48.3, 74.9) | 58.2 (44.3, 72.7) | 53.6 (37.9, 71.0) | <0.001 | <0.001 |
| Men, n (%) | 8159 | 4857 (59.53) | 1264 (61.96) | 1212 (59.47) | 1223 (59.92) | 1158 (56.76) | 0.002 | 0.002 |
| Body mass index (kg/m^2^) | 3201 | 23.9 (21.3, 26.7) | 24.2 (21.8, 27.1) | 23.8 (21.2, 26.6) | 23.8 (21.1, 26.7) | 23.3 (20.6, 26.3) | <0.001 | <0.001 |
| **Hospital service use at second S-Cre measurement, n (%)** | 8159 |  |  |  |  |  | <0.001 | − |
| Outpatient |  | 2379 (29.16) | 1074 (52.65) | 761 (37.34) | 437 (21.41) | 107 (5.25) |  |  |
| Emergency department |  | 1922 (23.56) | 423 (20.74) | 477 (23.41) | 506 (24.79) | 516 (25.29) |  |  |
| Inpatient |  | 3858 (47.29) | 543 (26.62) | 800 (39.25) | 1098 (53.80) | 1417 (69.46) |  |  |
| General ward |  | 1428 (37.01) | 255 (48.76) | 392 (46.34) | 447 (35.53) | 334 (27.13) |  |  |
| Intensive care unit |  | 2430 (62.99) | 268 (51.24) | 454 (53.66) | 811 (64.47) | 897 (72.87) |  |  |
| Fluid therapy between two measurements | 8159 | 4537 (55.61) | 602 (30.07) | 975 (45.62) | 1481 (63.78) | 1479 (87.10) | <0.001 | <0.001 |
| Fluid therapy on the index day | 8159 | 5779 (70.83) | 1026 (50.29) | 1297 (63.64) | 1572 (77.02) | 1884 (92.35) | <0.001 | <0.001 |
| **Comorbidities, n (%)** |  |  |  |  |  |  |  |  |
| Impaired kidney function | 8159 | 2650 (32.48) | 829 (40.64) | 713 (34.99) | 473 (23.17) | 635 (31.13) | <0.001 | <0.001 |
| Acute kidney failure (ICD-9 codes 584.5-584.9) | 8159 | 544 (6.67) | 154 (7.55) | 130 (6.38) | 112 (5.49) | 148 (7.25) | 0.473 | 0.473 |
| Diabetes mellitus | 8159 | 1990 (24.39) | 687 (33.68) | 520 (25.52) | 421 (20.63) | 362 (17.75) | <0.001 | <0.001 |
| Hypertension | 8159 | 2136 (26.18) | 797 (39.07) | 562 (27.58) | 464 (22.73) | 313 (15.34) | <0.001 | <0.001 |
| Noncancerous catastrophic illness status | 8159 | 1295 (15.87) | 286 (14.02) | 339 (16.63) | 311 (15.24) | 359 (17.60) | 0.010 | 0.010 |
| **Medication, n (%)** |  |  |  |  |  |  |  |  |
| Angiotensin-converting-enzyme inhibitors | 8159 | 1070 (13.11) | 324 (15.88) | 277 (13.59) | 271 (13.28) | 198 (9.71) | <0.001 | <0.001 |
| Angiotensin II receptor blockers | 8159 | 1384 (16.96) | 569 (27.89) | 377 (18.50) | 267 (13.08) | 171 (8.38) | <0.001 | <0.001 |
| Diuretics | 8159 | 2773 (33.99) | 800 (39.22) | 707 (34.69) | 671 (32.88) | 595 (29.17) | <0.001 | <0.001 |
| Oral hypoglycemic agents | 8159 | 1343 (16.46) | 484 (23.73) | 376 (18.45) | 282 (13.82) | 201 (9.85) | <0.001 | <0.001 |
| Insulin | 8159 | 1682 (20.62) | 506 (24.80) | 411 (20.17) | 377 (18.47) | 388 (19.02) | <0.001 | <0.001 |
| NSAIDs | 8159 | 2990 (36.65) | 633 (31.03) | 711 (34.89) | 819 (40.13) | 827 (40.54) | <0.001 | <0.001 |
| Radiocontrast | 8159 | 2710 (33.21) | 548 (26.86) | 653 (32.04) | 705 (34.54) | 804 (39.41) | <0.001 | <0.001 |
| **Lab data, median (IQR)** |  |  |  |  |  |  |  |  |
| Baseline BUN, mg/dL | 7382 | 16.0 (11.0, 33.0) | 20.0 (12.0, 47.5) | 17.5 (11.5, 35.0) | 15.0 (10.5, 26.3) | 14.5 (10.0, 26.5) | <0.001 | <0.001 |
| Second BUN, mg/dL | 5874 | 16.0 (10.0, 34.0) | 22.0 (12.0, 53.0) | 18.0 (12.0, 38.0) | 14.0 (10.0, 26.0) | 13.0 (9.0, 24.0) | <0.001 | <0.001 |
| Baseline serum creatinine, mg/dL | 8159 | 1.09 (0.82, 1.77) | 1.20 (0.88, 2.43) | 1.09 (0.82, 1.80) | 1.00 (0.80, 1.40) | 1.10 (0.80, 1.67) | <0.001 | <0.001 |
| Second serum creatinine, mg/dL | 8159 | 0.97 (0.73, 1.56) | 1.18 (0.87, 2.39) | 1.03 (0.78, 1.70) | 0.90 (0.72, 1.25) | 0.85 (0.61, 1.20) | <0.001 | <0.001 |
| Baseline eGFR, ml/min/1.73m^2^ | 8159 | 69.6 (35.2, 95.2) | 58.6 (22.8, 91.6) | 68.0 (32.4, 95.4) | 76.1 (47.3, 96.9) | 70.4 (39.6, 97.2) | <0.001 | <0.001 |
| Serum albumin, g/dL | 4825 | 3.40 (2.70, 4.00) | 3.70 (3.00, 4.30) | 3.50 (2.75, 4.20) | 3.30 (2.60, 3.90) | 3.00 (2.48, 3.60) | <0.001 | <0.001 |
| Hemoglobin, g/dL | 7422 | 12.4 (10.3, 14.2) | 12.1 (9.90, 14.2) | 12.2 (10.2, 14.1) | 12.6 (10.6, 14.3) | 12.4 (10.7, 14.1) | <0.001 | <0.001 |
| Sodium, mEq/L | 6780 | 138 (135, 140) | 138 (135, 140) | 138 (135, 140) | 138 (135, 141) | 138 (135, 140) | 0.002 | 0.003 |
| Potassium, mEq/L | 6983 | 3.80 (3.45, 4.25) | 4.00 (3.60, 4.50) | 3.90 (3.50, 4.35) | 3.70 (3.40, 4.10) | 3.65 (3.35, 4.05) | <0.001 | <0.001 |
| White blood cell count, 10^3^/μL | 7312 | 9.32 (6.65, 13.14) | 8.00 (6.00, 11.20) | 8.46 (6.20, 11.99) | 9.47 (6.95, 13.02) | 11.4 (8.03, 15.4) | <0.001 | <0.001 |
| C-reactive protein, mg/dL | 4958 | 1.54 (0.25, 7.26) | 1.03 (0.19, 5.19) | 1.42 (0.25, 6.56) | 1.71 (0.26, 7.30) | 2.23 (0.32, 10.49) | <0.001 | <0.001 |
| **Summary measures of baseline and second S-Cre, median (IQR)** |  |  |  |  |  |  |  |  |
| Time interval (hours) | 8159 | 5.72 (1.27, 9.55) | 1.53 (0.00, 6.41) | 4.21 (0.02, 8.80) | 6.32 (3.00, 10.13) | 7.93 (5.76, 11.27) | <0.001 | <0.001 |
| Difference (ΔS-Cre) | 8159 | 0.10 (0.04, 0.19) | 0.02 (0.01, 0.04) | 0.06 (0.04, 0.10) | 0.10 (0.08, 0.14) | 0.24 (0.16, 0.40) | <0.001 | <0.001 |
| Percent change (ΔS-Cre%) | 8159 | 7.48 (3.30, 14.14) | 1.79 (1.13, 2.56) | 5.13 (4.17, 6.23) | 10.0 (8.77, 11.9) | 20.9 (16.8, 27.7) | <0.001 | <0.001 |
| **Outcome, n (%)** |  |  |  |  |  |  |  |  |
| All-cause deaths before 2017-12-31 | 8159 | 3033 (37.17) | 692 (33.92) | 759 (37.24) | 778 (38.12) | 804 (39.41) | <0.001 | <0.001 |
| 3-year all-cause deaths | 8159 | 2145 (26.29) | 496 (24.31) | 536 (26.30) | 540 (26.46) | 573 (28.09) | 0.008 | 0.008 |
| 1-year all-cause deaths | 8159 | 1435 (17.59) | 305 (14.95) | 363 (17.81) | 372 (18.23) | 395 (19.36) | <0.001 | <0.001 |
| 30-day all-cause deaths | 8159 | 559 (6.85) | 99 (4.85) | 152 (7.46) | 134 (6.57) | 174 (8.53) | <0.001 | <0.001 |

^a^*p*-alues are calculated by Kruskal-wallis test for continuous variables and chi-square test for categorical variables.

^b^*p*-values for trend are calculated by Spearman’s correlation for continuous variables and by Cochran-Armitage trend test for binary variables.

**Abbreviations:** BUN, blood urea nitrogen; eGFR, estimated glomerular filtration rate; ICD, International Classification of Disease; NSAID, Nonsteroidal anti-inflammatory drugs; S-Cre, serum creatinine

**Supplementary Table 2a**. Baseline demographic and clinical characteristics based on quartiles of within-day serum creatinine difference (ΔS-Cre) among patients with deterioration kidney function (ΔS-Cre >0).

| **Variables** |  |  | **1st Quartile** | **2nd Quartile** | **3rd Quartile** | **4th Quartile** |  |  |
| --- | --- | --- | --- | --- | --- | --- | --- | --- |
| **\| ΔS-Cre \|** | **N** | **Total** | **<0.04** | **0.04≤~<0.09** | **0.09≤~<0.17** | ≥**0.17** | ***p*-value^a^** | ***p*-value for trend^b^** |
| **Sample size** |  | **6753** | **1683** | **1710** | **1677** | **1683** |  |  |
| **Demographics, median (IQR)** |  |  |  |  |  |  |  |  |
| Age, years | 6753 | 60.4 (46.6, 74.2) | 58.7 (46.3, 72.6) | 60.4 (47.0, 74.1) | 60.0 (44.8, 73.9) | 62.9 (48.4, 76.3) | <0.001 | <0.001 |
| Men, n (%) | 6753 | 3973 (58.83) | 943 (56.03) | 969 (56.67) | 977 (58.26) | 1084 (64.41) | <0.001 | <0.001 |
| Body mass index (kg/m^2^) | 2831 | 24.2 (21.3, 27.2) | 24.2 (21.3, 27.3) | 24.3 (21.3, 27.3) | 24.1 (21.3, 26.7) | 24.2 (21.4, 27.3) | 0.498 | 0.001 |
| **Hospital service use at second S-Cre measurement** | 6753 |  |  |  |  |  | <0.001 | − |
| Outpatient |  | 2015 (29.84) | 908 (53.95) | 684 (40.00) | 339 (20.21) | 84 (4.99) |  |  |
| Emergency department |  | 1527 (22.61) | 317 (18.84) | 363 (21.23) | 444 (26.48) | 403 (23.95) |  |  |
| Inpatient |  | 3211 (47.55) | 458 (27.21) | 663 (38.77) | 894 (53.31) | 1196 (71.06) |  |  |
| General ward |  | 1342 (41.79) | 195 (42.58) | 276 (41.63) | 386 (43.18) | 485 (40.55) |  |  |
| Intensive care unit |  | 1869 (58.21) | 263 (57.42) | 387 (58.37) | 508 (56.82) | 711 (59.45) |  |  |
| Fluid therapy between two measurements | 6753 | 3580 (53.01) | 547 (32.50) | 750 (43.86) | 986 (58.80) | 1297 (77.06) | <0.001 | <0.001 |
| Fluid therapy on the index day | 6753 | 4705 (69.67) | 831 (49.38) | 1053 (61.58) | 1276 (76.09) | 1545 (91.80) | <0.001 | <0.001 |
| **Comorbidities, n (%)** |  |  |  |  |  |  |  |  |
| Impaired kidney function | 6753 | 1829 (27.08) | 298 (17.71) | 368 (21.52) | 445 (26.54) | 718 (42.66) | <0.001 | <0.001 |
| Acute kidney failure (ICD-9 codes 584.5-584.9) | 6753 | 360 (5.33) | 59 (3.51) | 79 (4.62) | 76 (4.53) | 146 (8.67) | <0.001 | <0.001 |
| Diabetes mellitus | 6753 | 1798 (26.63) | 451 (26.80) | 484 (28.30) | 399 (23.79) | 464 (27.57) | 0.646 | 0.646 |
| Hypertension | 6753 | 2061 (30.52) | 504 (29.95) | 561 (32.81) | 465 (27.73) | 531 (31.55) | 0.954 | 0.954 |
| Noncancerous catastrophic illness status | 6753 | 1161 (17.19) | 261 (15.51) | 340 (19.88) | 256 (15.27) | 304 (18.06) | 0.464 | 0.464 |
| **Medication, n (%)** |  |  |  |  |  |  |  |  |
| Angiotensin-converting-enzyme inhibitors | 6753 | 1094 (16.20) | 201 (11.94) | 253 (14.80) | 286 (17.05) | 354 (21.03) | <0.001 | <0.001 |
| Angiotensin II receptor blockers | 6753 | 1317 (19.50) | 364 (21.63) | 370 (21.64) | 282 (16.82) | 301 (17.88) | <0.001 | <0.001 |
| Diuretics | 6753 | 2683 (39.73) | 470 (27.93) | 629 (36.78) | 650 (38.76) | 934 (55.50) | <0.001 | <0.001 |
| Oral hypoglycemic agents | 6753 | 1281 (18.97) | 347 (20.62) | 357 (20.88) | 290 (17.29) | 287 (17.05) | 0.001 | 0.001 |
| Insulin | 6753 | 1510 (22.36) | 284 (16.87) | 345 (20.18) | 354 (21.11) | 527 (31.31) | <0.001 | <0.001 |
| NSAIDs | 6753 | 2923 (43.28) | 628 (37.31) | 659 (38.54) | 781 (46.57) | 855 (50.80) | <0.001 | <0.001 |
| Radiocontrast | 6753 | 2146 (31.78) | 498 (29.59) | 555 (32.46) | 499 (29.76) | 594 (35.29) | 0.005 | 0.005 |
| **Lab data, median (IQR)** |  |  |  |  |  |  |  |  |
| Baseline BUN, mg/dL | 6103 | 16.5 (11.0, 31.0) | 14.0 (10.0, 22.0) | 15.0 (11.0, 25.0) | 16.0 (11.0, 32.0) | 23.0 (14.5, 43.8) | <0.001 | <0.001 |
| Second BUN, mg/dL | 4875 | 17.0 (11.0, 34.0) | 14.0 (10.0, 23.0) | 15.0 (11.0, 25.0) | 16.0 (11.0, 35.0) | 25.0 (15.0, 47.0) | <0.001 | <0.001 |
| Baseline serum creatinine, mg/dL | 6753 | 0.96 (0.73, 1.53) | 0.88 (0.69, 1.20) | 0.92 (0.70, 1.31) | 0.94 (0.72, 1.52) | 1.29 (0.87, 2.23) | <0.001 | <0.001 |
| Second serum creatinine, mg/dL | 6753 | 1.09 (0.82, 1.76) | 0.91 (0.71, 1.22) | 0.98 (0.76, 1.37) | 1.08 (0.84, 1.65) | 1.69 (1.16, 2.80) | <0.001 | <0.001 |
| Baseline eGFR, ml/min/1.73m^2^ | 6753 | 78.6 (41.2, 100.9) | 88.0 (57.1, 104.5) | 83.3 (48.7, 102.7) | 81.1 (41.8, 102.2) | 54.4 (26.4, 88.1) | <0.001 | <0.001 |
| Serum albumin, g/dL | 3938 | 3.20 (2.60, 3.90) | 3.75 (3.00, 4.40) | 3.60 (2.80, 4.20) | 3.10 (2.50, 3.70) | 2.90 (2.30, 3.40) | <0.001 | <0.001 |
| Hemoglobin, g/dL | 6087 | 12.3 (10.3, 14.0) | 12.8 (10.8, 14.3) | 12.4 (10.4, 14.1) | 12.2 (10.2, 14.0) | 11.7 (9.8, 13.6) | <0.001 | <0.001 |
| Sodium, mEq/L | 5625 | 138 (135, 140) | 138 (135, 140) | 138 (136, 140) | 138 (135, 140) | 138 (134, 141) | 0.182 | 0.005 |
| Potassium, mEq/L | 5861 | 3.83 (3.45, 4.30) | 3.90 (3.50, 4.20) | 3.85 (3.50, 4.30) | 3.75 (3.40, 4.20) | 3.85 (3.45, 4.40) | <0.001 | 0.074 |
| White blood cell count, 10^3^/μL | 6000 | 9.23 (6.70, 13.05) | 7.75 (6.05, 10.65) | 8.70 (6.43, 12.23) | 9.52 (6.91, 13.22) | 11.15 (8.03, 15.33) | <0.001 | <0.001 |
| C-reactive protein, mg/dL | 4230 | 1.84 (0.32, 7.09) | 0.72 (0.16, 4.73) | 1.27 (0.25, 5.23) | 2.06 (0.38, 7.54) | 3.15 (0.70, 10.49) | <0.001 | <0.001 |
| **Summary measures of baseline and second S-Cre** |  |  |  |  |  |  |  |  |
| Time interval (hours) | 6753 | 6.22 (1.10, 10.90) | 1.63 (0.00, 7.42) | 4.13 (0.02, 9.27) | 7.00 (3.15, 11.52) | 9.28 (6.18, 13.45) | <0.001 | <0.001 |
| Difference (ΔS-Cre) | 6753 | 0.09 (0.04, 0.17) | 0.02 (0.01, 0.03) | 0.06 (0.05, 0.07) | 0.10 (0.10, 0.13) | 0.30 (0.20, 0.46) | <0.001 | <0.001 |
| Percent change (ΔS-Cre%) | 6753 | 7.69 (3.25, 15.79) | 2.04 (1.16, 3.33) | 6.28 (4.10, 8.89) | 11.81 (7.52, 16.22) | 25.0 (14.3, 37.8) | <0.001 | <0.001 |
| **Outcome** |  |  |  |  |  |  |  |  |
| All-cause deaths before 2017-12-31 | 6753 | 2733 (40.47) | 437 (25.97) | 530 (30.99) | 752 (44.84) | 1014 (60.25) | <0.001 | <0.001 |
| 3-year all-cause deaths | 6753 | 2021 (29.93) | 313 (18.60) | 400 (23.39) | 527 (31.43) | 781 (46.41) | <0.001 | <0.001 |
| 1-year all-cause deaths | 6753 | 1459 (21.61) | 193 (11.47) | 272 (15.91) | 387 (23.08) | 607 (36.07) | <0.001 | <0.001 |
| 30-day all-cause deaths | 6753 | 745 (11.03) | 74 (4.40) | 103 (6.02) | 183 (10.91) | 385 (22.88) | <0.001 | <0.001 |

^a^*p*-alues are calculated by Kruskal-wallis test for continuous variables and chi-square test for categorical variables.

^b^*p*-values for trend are calculated by Spearman’s correlation for continuous variables and by Cochran-Armitage trend test for binary variables.

**Abbreviations:** BUN, blood urea nitrogen; eGFR, estimated glomerular filtration rate; ICD, International Classification of Disease; NSAID, Nonsteroidal anti-inflammatory drugs; S-Cre, serum creatinine

**Supplementary Table 2b.** Baseline demographic and clinical characteristics based on quartiles of within-day serum creatinine difference (ΔS-Cre) among patients with improving kidney function (ΔS-Cre <0).

| **Variables** |  |  | **1st Quartile** | **2nd Quartile** | **3rd Quartile** | **4th Quartile** |  |  |
| --- | --- | --- | --- | --- | --- | --- | --- | --- |
| **\| ΔS-Cre \|** | **N** | **Total** | **< 0.04** | **0.04≤~<0.10** | **0.10≤~<0.18** | ≥**0.18** | ***p* -value^a^** | ***p* -value for trend^b^** |
| **Sample size** |  | **8159** | **1960** | **2003** | **2084** | **2112** |  |  |
| **Demographics, median (IQR)** |  |  |  |  |  |  |  |  |
| Age, years | 8159 | 59.0 (44.8, 73.3) | 58.0 (45.0, 71.2) | 58.4 (45.7, 72.1) | 59.4 (43.5, 74.0) | 61.3 (45.4, 75.3) | <0.001 | <0.001 |
| Men, n (%) | 8159 | 4857 (59.53) | 1110 (56.63) | 1153 (57.56) | 1247 (59.84) | 1347 (63.78) | <0.001 | <0.001 |
| Body mass index (kg/m^2^) | 3201 | 23.9 (21.3, 26.7) | 24.2 (21.6, 27.2) | 24.0 (21.3, 26.9) | 23.5 (21.3, 26.4) | 23.5 (20.6, 26.4) | <0.001 | 0.001 |
| **Hospital service use at second S-Cre measurement, n (%)** | 8159 |  |  |  |  |  | <0.001 | − |
| Outpatient |  | 2379 (29.16) | 1115 (56.89) | 824 (41.14) | 382 (18.33) | 58 (2.75) |  |  |
| Emergency department |  | 1922 (23.56) | 325 (16.58) | 427 (21.32) | 571 (27.40) | 599 (28.36) |  |  |
| Inpatient |  | 3858 (47.29) | 520 (26.53) | 752 (37.54) | 1131 (54.27) | 1455 (68.89) |  |  |
| General ward |  | 1428 (37.01) | 222 (42.69) | 302 (40.16) | 429 (37.93) | 475 (32.65) |  |  |
| Intensive care unit |  | 2430 (62.99) | 298 (57.31) | 450 (59.84) | 702 (62.07) | 980 (67.35) |  |  |
| Fluid therapy between two measurements | 8159 | 4537 (55.61) | 572 (29.18) | 878 (43.83) | 1277 (62.54) | 1810 (84.03) | <.0001 | <.0001 |
| Fluid therapy on the index day | 8159 | 5779 (70.83) | 945 (48.21) | 1227 (61.26) | 1649 (79.13) | 1958 (92.71) | <.0001 | <.0001 |
| **Comorbidities, n (%)** |  |  |  |  |  |  |  |  |
| Impaired kidney function | 8159 | 2650 (32.48) | 313 (15.97) | 435 (21.72) | 606 (29.08) | 1296 (61.36) | <0.001 | <0.001 |
| Acute kidney failure (ICD-9 codes 584.5-584.9) | 8159 | 544 (6.67) | 67 (3.42) | 72 (3.59) | 98 (4.70) | 307 (14.54) | <0.001 | <0.001 |
| Diabetes mellitus | 8159 | 1990 (24.39) | 492 (25.10) | 484 (24.16) | 462 (22.17) | 552 (26.14) | 0.755 | 0.202 |
| Hypertension | 8159 | 2136 (26.18) | 563 (28.72) | 545 (27.21) | 527 (25.29) | 501 (23.72) | <0.001 | 0.002 |
| Noncancerous catastrophic illness status | 8159 | 1295 (15.87) | 316 (16.12) | 347 (17.32) | 326 (15.64) | 306 (14.49) | 0.065 | 0.265 |
| **Medication, n (%)** |  |  |  |  |  |  |  |  |
| Angiotensin-converting-enzyme inhibitors | 8159 | 1070 (13.11) | 229 (11.68) | 222 (11.08) | 325 (15.60) | 294 (13.92) | <0.001 | 0.004 |
| Angiotensin II receptor blockers | 8159 | 1384 (16.96) | 407 (20.77) | 365 (18.22) | 319 (15.31) | 293 (13.87) | <0.001 | <0.001 |
| Diuretics | 8159 | 2773 (33.99) | 504 (25.71) | 606 (30.25) | 765 (36.71) | 898 (42.52) | <0.001 | <0.001 |
| Oral hypoglycemic agents | 8159 | 1343 (16.46) | 382 (19.49) | 335 (16.72) | 315 (15.12) | 311 (14.73) | <0.001 | <0.001 |
| Insulin | 8159 | 1682 (20.62) | 293 (14.95) | 350 (17.47) | 398 (19.10) | 641 (30.35) | <0.001 | <0.001 |
| NSAIDs | 8159 | 2990 (36.65) | 650 (33.16) | 713 (35.60) | 833 (39.97) | 794 (37.59) | <0.001 | 0.002 |
| Radiocontrast | 8159 | 2710 (33.21) | 626 (31.94) | 694 (34.65) | 633 (30.37) | 757 (35.84) | 0.106 | 0.009 |
| **Lab data, median (IQR)** |  |  |  |  |  |  |  |  |
| Baseline BUN, mg/dL | 7382 | 16.0 (11.0, 33.0) | 14.0 (10.0, 20.0) | 14.0 (10.0, 23.0) | 16.0 (11.0, 30.0) | 28.0 (14.0, 56.5) | <0.001 | <0.001 |
| Second BUN, mg/dL | 5874 | 16.0 (10.0, 34.0) | 14.0 (10.0, 21.0) | 14.0 (10.0, 24.0) | 15.0 (10.0, 30.0) | 25.0 (12.0, 54.0) | <0.001 | <0.001 |
| Baseline S-Cre, mg/dL | 8159 | 1.09 (0.82, 1.77) | 0.91 (0.71, 1.18) | 0.96 (0.76, 1.32) | 1.07 (0.84, 1.60) | 1.80 (1.18, 3.15) | <0.001 | <0.001 |
| Second S-Cre, mg/dL | 8159 | 0.97 (0.73, 1.56) | 0.89 (0.69, 1.16) | 0.90 (0.70, 1.26) | 0.94 (0.71, 1.50) | 1.35 (0.90, 2.60) | <0.001 | <0.001 |
| Baseline eGFR, ml/min/1.73m^2^ | 8159 | 69.6 (35.2, 95.2) | 86.8 (58.9, 102.8) | 81.5 (50.5, 99.9) | 70.8 (38.3, 94.4) | 36.4 (17.3, 65.7) | <0.001 | <0.001 |
| Serum albumin, g/dL | 4825 | 3.40 (2.70, 4.00) | 4.00 (3.20, 4.50) | 3.60 (3.00, 4.30) | 3.20 (2.60, 3.80) | 2.90 (2.30, 3.53) | <0.001 | <0.001 |
| Hemoglobin, g/dL | 7422 | 12.4 (10.3, 14.2) | 12.9 (11.1, 14.5) | 12.6 (10.7, 14.3) | 12.2 (10.2, 14.0) | 11.8 (9.7, 13.7) | <0.001 | <0.001 |
| Sodium, mEq/L | 6780 | 138 (135, 140) | 138 (136, 140) | 138 (136, 140) | 138 (135, 140) | 138 (134, 141) | 0.143 | 0.007 |
| Potassium, mEq/L | 6983 | 3.80 (3.45, 4.25) | 3.90 (3.53, 4.25) | 3.80 (3.45, 4.30) | 3.73 (3.40, 4.15) | 3.80 (3.40, 4.35) | <0.001 | 0.085 |
| White blood cell count, 10^3^/μL | 7312 | 9.32 (6.65, 13.14) | 7.69 (5.90, 10.65) | 8.57 (6.21, 11.85) | 9.54 (6.95, 13.12) | 11.55 (8.01, 15.9) | <0.001 | <0.001 |
| C-reactive protein, mg/dL | 4958 | 1.54 (0.25, 7.26) | 0.57 (0.13, 3.91) | 0.89 (0.16, 4.86) | 1.67 (0.29, 6.61) | 3.50 (0.56, 13.83) | <0.001 | <0.001 |
| **Summary measures of baseline and second S-Cre, median (IQR)** |  |  |  |  |  |  |  |  |
| Time interval (hours) | 8159 | 5.72 (1.27, 9.55) | 1.09 (0.00, 6.25) | 3.73 (0.02, 8.47) | 6.35 (3.20, 9.96) | 8.03 (5.82, 11.32) | <0.001 | <0.001 |
| Difference (ΔS-Cre) | 8159 | 0.10 (0.04, 0.19) | 0.02 (0.01, 0.03) | 0.06 (0.05, 0.08) | 0.10 (0.10, 0.13) | 0.30 (0.21, 0.47) | <0.001 | <0.001 |
| Percent change (ΔS-Cre%) | 8159 | 7.48 (3.30, 14.14) | 2.06 (1.17, 3.27) | 6.17 (4.19, 8.43) | 10.3 (7.14, 14.3) | 19.3 (11.8, 27.3) | <0.001 | <0.001 |
| **Outcome, n (%)** |  |  |  |  |  |  |  |  |
| All-cause deaths before 2017-12-31 | 8159 | 3033 (37.17) | 461 (23.52) | 569 (28.41) | 903 (43.33) | 1100 (52.08) | <0.001 | <0.001 |
| 3-year all-cause deaths | 8159 | 2145 (26.29) | 341 (17.40) | 421 (21.02) | 604 (28.98) | 779 (36.88) | <0.001 | <0.001 |
| 1-year all-cause deaths | 8159 | 1435 (17.59) | 209 (10.66) | 266 (13.28) | 407 (19.53) | 553 (26.18) | <0.001 | <0.001 |
| 30-day all-cause deaths | 8159 | 559 (6.85) | 60 (3.06) | 95 (4.74) | 151 (7.25) | 253 (11.98) | <0.001 | <0.001 |

^a^*p*-alues are calculated by Kruskal-wallis test for continuous variables and chi-square test for categorical variables.

^b^*p*-values for trend are calculated by Spearman’s correlation for continuous variables and by Cochran-Armitage trend test for binary variables.

**Abbreviations:** BUN, blood urea nitrogen; eGFR, estimated glomerular filtration rate; ICD, International Classification of Disease; NSAID, Nonsteroidal anti-inflammatory drugs; S-Cre, serum creatinine

**Supplementary Table 3.** Hazard ratios (95% confidence interval) of 1-year (for Groups 1 and 2) and 30-day (for Groups 3 and 4) all-cause mortality according to every 0.1 mg/dL change in S-Cre levels repeated within 24 hours. ED, emergency department; INPT, inpatient; OPT, outpatient; S-Cre, serum creatinine.

|  |  |  |  | **Model 1** |  | **Model 2** |  | **Model 3** |  |
| --- | --- | --- | --- | --- | --- | --- | --- | --- | --- |
|  | **Case/N** | **Mortality (%)** | **Crude HR** | **Adjusted HR** | ***p*-value** | **Adjusted HR** | ***p*-value** | **Adjusted HR** | ***p*-value** |
|  |  |  | **(95% CI)** | **(95% CI)** |  | **(95% CI)** |  | **(95% CI)** |  |
| **1-year mortality** |  |  |  |  |  |  |  |  |  |
| Overall | 2894/14912 | 19.4% | 1.09 (1.08, 1.10) | 1.05 (1.04, 1.07) | <0.001 | 1.03 (1.02, 1.04) | < 0.001 | 1.02 (1.00, 1.03) | 0.039 |
| Deteriorating | 1459/6753 | 21.6% | 1.16 (1.15, 1.18) | 1.14 (1.12, 1.16) | <0.001 | 1.1 (1.08, 1.12) | < 0.001 | 1.09 (1.07, 1.11) | < 0.001 |
| Improving | 1435/8159 | 17.6% | 1.06 (1.05, 1.08) | 1.00 (0.98, 1.02) | 0.804 | 0.97 (0.95, 1.00) | 0.022 | 0.96 (0.94, 0.98) | 0.001 |
| **Group 1 (OPT to OPT)** | |  |  |  |  |  |  |  |  |
| Overall | 187/4145 | 4.5% | 1.21 (1.05, 1.40) | 1.14 (0.97, 1.34) | 0.101 | 1.16 (0.98, 1.38) | 0.089 | 1.08 (0.88, 1.32) | 0.469 |
| Deteriorating | 95/1882 | 5.0% | 1.15 (0.92, 1.43) | 1.12 (0.89, 1.40) | 0.348 | 1.16 (0.88, 1.52) | 0.284 | 1.06 (0.76, 1.47) | 0.728 |
| Improving | 92/2263 | 4.1% | 1.28 (1.02, 1.61) | 1.17 (0.90, 1.52) | 0.234 | 1.16 (0.89, 1.51) | 0.268 | 1.13 (0.85, 1.51) | 0.409 |
| **Group 2 (OPT to ED or INPT)** | |  |  |  |  |  |  |  |  |
| Overall | 244/1761 | 13.9% | 1.06 (0.97, 1.15) | 1.00 (0.90, 1.10) | 0.945 | 0.98 (0.88, 1.08) | 0.664 | 0.96 (0.86, 1.06) | 0.420 |
| Deteriorating | 102/782 | 13.0% | 1.14 (1.01, 1.28) | 1.09 (0.96, 1.23) | 0.193 | 1.09 (0.95, 1.24) | 0.216 | 1.06 (0.92, 1.23) | 0.435 |
| Improving | 142/979 | 14.5% | 0.95 (0.81, 1.13) | 0.86 (0.70, 1.06) | 0.161 | 0.79 (0.64, 0.99) | 0.038 | 0.80 (0.64, 0.99) | 0.040 |
| **30-day mortality** |  |  |  |  |  |  |  |  |  |
| Overall | 1304/14912 | 8.7% | 1.14 (1.12, 1.15) | 1.10 (1.08, 1.12) | <0.001 | 1.07 (1.05, 1.09) | < 0.001 | 1.06 (1.04, 1.08) | < 0.001 |
| Deteriorating | 745/6753 | 11.0% | 1.26 (1.22, 1.30) | 1.23 (1.19, 1.27) | <0.001 | 1.17 (1.14, 1.21) | < 0.001 | 1.16 (1.12, 1.20) | < 0.001 |
| Improving | 559/8159 | 6.9% | 1.10 (1.07, 1.13) | 1.04 (1.00, 1.07) | 0.025 | 1.00 (0.96, 1.03) | 0.903 | 0.99 (0.95, 1.03) | 0.598 |
| **Group 3 (ED to ED or INPT)** | |  |  |  |  |  |  |  |  |
| Overall | 604/5545 | 10.9% | 1.07 (1.04, 1.09) | 1.03 (1.01, 1.06) | 0.011 | 1.04 (1.01, 1.06) | 0.008 | 1.04 (1.01, 1.07) | 0.006 |
| Deteriorating | 270/2184 | 12.4% | 1.17 (1.10, 1.24) | 1.15 (1.09, 1.23) | <.0001 | 1.17 (1.10, 1.25) | < 0.001 | 1.19 (1.11, 1.27) | < 0.001 |
| Improving | 334/3361 | 9.9% | 1.05 (1.01, 1.09) | 0.99 (0.95, 1.04) | 0.729 | 0.99 (0.95, 1.04) | 0.695 | 1.00 (0.95, 1.04) | 0.850 |
| **Group 4 (INPT to INPT)** | |  |  |  |  |  |  |  |  |
| Overall | 634/3164 | 20.0% | 1.12 (1.08, 1.16) | 1.09 (1.06, 1.13) | <.0001 | 1.08 (1.05, 1.12) | < 0.001 | 1.08 (1.03, 1.12) | < 0.001 |
| Deteriorating | 437/1736 | 25.2% | 1.15 (1.10, 1.21) | 1.14 (1.08, 1.19) | <.0001 | 1.14 (1.08, 1.2) | < 0.001 | 1.14 (1.08, 1.21) | < 0.001 |
| Improving | 197/1428 | 13.8% | 1.07 (0.94, 1.21) | 0.97 (0.84, 1.12) | 0.646 | 0.92 (0.79, 1.07) | 0.297 | 0.92 (0.77, 1.08) | 0.300 |

**Model 1:** Adjusted for gender, body mass index, diabetes, hypertension, impaired kidney function, noncancerous catastrophic illness, acute kidney failure, baseline eGFR.

**Model 2:** Further adjusted for medications listed in Table 1 including fluid therapy between two S-Cre measurements.

**Model 3:** Further adjusted for baseline blood urea nitrogen, C-reactive protein, white blood cell count, serum albumin, hemoglobin.

**Supplementary Table 4.** Hazard ratios (95% confidence interval) of 3-year all-cause mortality according to every 5% change in S-Cre levels repeated within 24 hours. ED, emergency department; INPT, inpatient; OPT, outpatient; S-Cre, serum creatinine.

|  |  |  |  | **Model 1** |  | **Model 2** |  | **Model 3** |  |
| --- | --- | --- | --- | --- | --- | --- | --- | --- | --- |
|  | **Case/N** | **Mortality (%)** | **Crude HR** | **Adjusted HR** | ***p*-value** | **Adjusted HR** | ***p* -value** | **Adjusted HR** | ***p* -value** |
|  |  |  | **(95% CI)** | **(95% CI)** |  | **(95% CI)** |  | **(95% CI)** |  |
| **3-year mortality** |  |  |  |  |  |  |  |  |  |
| Overall | 4166/14912 | 27.9% | 1.03 (1.03, 1.04) | 1.05 (1.04, 1.05) | <0.001 | 1.03 (1.02, 1.04) | < 0.001 | 1.02 (1.01, 1.03) | < 0.001 |
| Deteriorating | 2021/6753 | 29.9% | 1.03 (1.02, 1.04) | 1.05 (1.04, 1.06) | <0.001 | 1.04 (1.03, 1.05) | < 0.001 | 1.03 (1.02, 1.04) | < 0.001 |
| Improving | 2145/8159 | 26.3% | 1.05 (1.03, 1.07) | 1.02 (1.00, 1.04) | 0.067 | 0.97 (0.95, 0.99) | 0.011 | 0.96 (0.94, 0.99) | 0.005 |
| **Group 1 (OPT to OPT)** | |  |  |  |  |  |  |  |  |
| Overall | 423/4145 | 10.2% | 0.92 (0.81, 1.04) | 1.10 (0.97, 1.24) | 0.128 | 1.08 (0.96, 1.22) | 0.210 | 1.05 (0.93, 1.19) | 0.440 |
| Deteriorating | 215/1882 | 11.4% | 0.99 (0.86, 1.15) | 1.14 (0.99, 1.32) | 0.061 | 1.13 (0.97, 1.31) | 0.111 | 1.08 (0.93, 1.26) | 0.285 |
| Improving | 208/2263 | 9.2% | 0.78 (0.63, 0.97) | 0.97 (0.77, 1.22) | 0.790 | 0.98 (0.78, 1.24) | 0.884 | 0.96 (0.76, 1.22) | 0.760 |
| **Group 2 (OPT to ED or INPT)** | |  |  |  |  |  |  |  |  |
| Overall | 410/1761 | 23.3% | 0.99 (0.94, 1.05) | 1.05 (1.00, 1.10) | 0.042 | 1.05 (1.00, 1.11) | 0.033 | 1.05 (1.00, 1.1) | 0.074 |
| Deteriorating | 176/782 | 22.5% | 1.01 (0.95, 1.08) | 1.07 (1.01, 1.14) | 0.019 | 1.08 (1.02, 1.15) | 0.010 | 1.06 (0.99, 1.13) | 0.074 |
| Improving | 234/979 | 23.9% | 0.96 (0.86, 1.08) | 1.04 (0.92, 1.16) | 0.553 | 0.99 (0.89, 1.11) | 0.887 | 1.01 (0.9, 1.14) | 0.872 |
| **Group 3 (ED to ED or INPT)** | |  |  |  |  |  |  |  |  |
| Overall | 1719/5545 | 31.0% | 1.01 (0.99, 1.02) | 1.01 (1.00, 1.03) | 0.167 | 1.01 (1.00, 1.03) | 0.060 | 1.02 (1.00, 1.03) | 0.036 |
| Deteriorating | 662/2184 | 30.3% | 1.03 (1.01, 1.04) | 1.04 (1.03, 1.05) | <0.001 | 1.04 (1.03, 1.05) | < 0.001 | 1.03 (1.02, 1.05) | < 0.001 |
| Improving | 1057/3361 | 31.4% | 0.93 (0.90, 0.96) | 0.91 (0.89, 0.94) | <0.001 | 0.92 (0.89, 0.95) | < 0.001 | 0.92 (0.89, 0.95) | < 0.001 |
| **Group 4 (INPT to INPT)** | |  |  |  |  |  |  |  |  |
| Overall | 1563/3164 | 49.4% | 1.01 (1.00, 1.02) | 1.02 (1.01, 1.03) | <0.001 | 1.02 (1.01, 1.03) | 0.001 | 1.01 (1.00, 1.02) | 0.051 |
| Deteriorating | 937/1736 | 54.0% | 1.00 (0.99, 1.01) | 1.02 (1.01, 1.03) | <0.001 | 1.02 (1.01, 1.03) | 0.001 | 1.02 (1.00, 1.03) | 0.012 |
| Improving | 626/1428 | 43.8% | 0.96 (0.92, 1.01) | 0.98 (0.93, 1.03) | 0.374 | 0.96 (0.91, 1.01) | 0.134 | 0.97 (0.92, 1.03) | 0.304 |

**Model 1:** Adjusted for gender, body mass index, diabetes, hypertension, impaired kidney function, noncancerous catastrophic illness, acute kidney failure, baseline eGFR.

**Model 2:** Further adjusted for medications listed in Table 1 including fluid therapy between two S-Cre measurements.

**Model 3:** Further adjusted for baseline blood urea nitrogen, C-reactive protein, white blood cell count, serum albumin, hemoglobin.

**Supplementary Table 5.** Hazard ratios (95% confidence interval) of 3-year all-cause mortality according to every 0.1 mg/dL change in S-Cre levels repeated within 24 hours. ED, emergency department; INPT, inpatient; OPT, outpatient; S-Cre, serum creatinine.

|  |  |  |  | **Model 1** |  | **Model 2** |  | **Model 3** |  |
| --- | --- | --- | --- | --- | --- | --- | --- | --- | --- |
|  | **Case/N** | **Mortality (%)** | **Crude HR** | **Adjusted HR** | ***p*-value** | **Adjusted HR** | ***p*-value** | **Adjusted HR** | ***p*-value** |
|  |  |  | **(95% CI)** | **(95% CI)** |  | **(95% CI)** |  | **(95% CI)** |  |
| **3-year mortality** |  |  |  |  |  |  |  |  |  |
| Overall | 4166/14912 | 27.9% | 1.08 (1.07, 1.09) | 1.04 (1.03, 1.05) | <0.001 | 1.01 (1.00, 1.02) | 0.045 | 1.00 (0.99, 1.01) | 0.861 |
| Deteriorating | 2021/6753 | 29.9% | 1.14 (1.12, 1.15) | 1.11 (1.10, 1.13) | <0.001 | 1.07 (1.06, 1.09) | < 0.001 | 1.06 (1.04, 1.08) | < 0.001 |
| Improving | 2145/8159 | 26.3% | 1.05 (1.04, 1.07) | 0.99 (0.98, 1.01) | 0.356 | 0.96 (0.95, 0.98) | < 0.001 | 0.96 (0.94, 0.98) | < 0.001 |
| **Group 1 (OPT to OPT)** | |  |  |  |  |  |  |  |  |
| Overall | 423/4145 | 10.2% | 1.19 (1.06, 1.33) | 1.07 (0.93, 1.22) | 0.350 | 1.09 (0.94, 1.25) | 0.249 | 1.04 (0.9, 1.21) | 0.603 |
| Deteriorating | 215/1882 | 11.4% | 1.19 (1.03, 1.37) | 1.12 (0.97, 1.30) | 0.131 | 1.17 (0.99, 1.37) | 0.063 | 1.13 (0.94, 1.35) | 0.196 |
| Improving | 208/2263 | 9.2% | 1.18 (0.95, 1.47) | 0.96 (0.74, 1.24) | 0.751 | 0.99 (0.76, 1.27) | 0.916 | 0.94 (0.72, 1.23) | 0.654 |
| **Group 2 (OPT to ED or INPT)** | |  |  |  |  |  |  |  |  |
| Overall | 410/1761 | 23.3% | 1.04 (0.97, 1.11) | 0.99 (0.91, 1.07) | 0.739 | 0.97 (0.9, 1.05) | 0.524 | 0.96 (0.89, 1.04) | 0.347 |
| Deteriorating | 176/782 | 22.5% | 1.12 (1.02, 1.23) | 1.08 (0.98, 1.20) | 0.136 | 1.1 (0.99, 1.22) | 0.073 | 1.07 (0.95, 1.19) | 0.260 |
| Improving | 234/979 | 23.9% | 0.96 (0.85, 1.08) | 0.89 (0.77, 1.03) | 0.113 | 0.84 (0.72, 0.97) | 0.018 | 0.84 (0.73, 0.98) | 0.025 |
| **Group 3 (ED to ED or INPT)** | |  |  |  |  |  |  |  |  |
| Overall | 1719/5545 | 31.0% | 1.03 (1.02, 1.05) | 0.98 (0.96, 0.99) | 0.009 | 0.98 (0.96, 1.00) | 0.040 | 0.98 (0.96, 1) | 0.053 |
| Deteriorating | 662/2184 | 30.3% | 1.08 (1.05, 1.10) | 1.06 (1.03, 1.09) | <0.001 | 1.06 (1.03, 1.09) | < 0.001 | 1.05 (1.02, 1.09) | < 0.001 |
| Improving | 1057/3361 | 31.4% | 1.02 (1.00, 1.03) | 0.94 (0.92, 0.96) | <0.001 | 0.94 (0.92, 0.97) | < 0.001 | 0.94 (0.92, 0.97) | < 0.001 |
| **Group 4 (INPT to INPT)** | |  |  |  |  |  |  |  |  |
| Overall | 1563/3164 | 49.4% | 1.05 (1.03, 1.06) | 1.03 (1.01, 1.05) | 0.008 | 1.02 (1, 1.04) | 0.053 | 1.01 (0.99, 1.03) | 0.258 |
| Deteriorating | 937/1736 | 54.0% | 1.07 (1.05, 1.10) | 1.05 (1.03, 1.08) | <0.001 | 1.05 (1.02, 1.08) | < 0.001 | 1.05 (1.03, 1.08) | < 0.001 |
| Improving | 626/1428 | 43.8% | 0.99 (0.96, 1.03) | 0.96 (0.92, 1.00) | 0.065 | 0.95 (0.91, 0.99) | 0.025 | 0.94 (0.9, 0.99) | 0.009 |

**Model 1:** Adjusted for gender, body mass index, diabetes, hypertension, impaired kidney function, noncancerous catastrophic illness, acute kidney failure, baseline eGFR.

**Model 2:** Further adjusted for medications listed in Table 1 including fluid therapy between two S-Cre measurements.

**Model 3:** Further adjusted for baseline blood urea nitrogen, C-reactive protein, white blood cell count, serum albumin, hemoglobin.

**Supplementary Table 6.** Sensitivity analysis of 3-year all-cause mortality according to every 5% change in S-Cre levels repeated within 24 hours after additionally adjusted for time intervals. ED, emergency department; INPT, inpatient; OPT, outpatient; S-Cre, serum creatinine.

|  |  |  | **Original Model 3** |  | **Further adjusted for time intervals between S-Cre measurements** |  |
| --- | --- | --- | --- | --- | --- | --- |
|  | **Case/N** | **Mortality (%)** | **Adjusted HR** | ***p*-value** | **Adjusted HR** | ***p*-value** |
|  |  |  | **(95% CI)** |  | **(95% CI)** |  |
| **3-year mortality** |  |  |  |  |  |  |
| Overall | 4166/14912 | 27.9% | 1.02 (1.01, 1.03) | < 0.001 | 1.02 (1.01, 1.03) | < 0.001 |
| Deteriorating | 2021/6753 | 29.9% | 1.03 (1.02, 1.04) | < 0.001 | 1.03 (1.02, 1.03) | < 0.001 |
| Improving | 2145/8159 | 26.3% | 0.96 (0.94, 0.99) | 0.005 | 0.96 (0.94, 0.98) | 0.0017 |
| **Group 1 (OPT to OPT)** | |  |  |  |  |  |
| Overall | 423/4145 | 10.2% | 1.05 (0.93, 1.19) | 0.440 | 1.06 (0.94, 1.20) | 0.342 |
| Deteriorating | 215/1882 | 11.4% | 1.08 (0.93, 1.26) | 0.285 | 1.08 (0.92, 1.26) | 0.338 |
| Improving | 208/2263 | 9.2% | 0.96 (0.76, 1.22) | 0.760 | 0.98 (0.77, 1.25) | 0.884 |
| **Group 2 (OPT to ED or INPT)** | |  |  |  |  |  |
| Overall | 410/1761 | 23.3% | 1.05 (1.00, 1.1) | 0.074 | 1.05 (1.00, 1.11) | 0.065 |
| Deteriorating | 176/782 | 22.5% | 1.06 (0.99, 1.13) | 0.074 | 1.06 (0.99, 1.14) | 0.072 |
| Improving | 234/979 | 23.9% | 1.01 (0.9, 1.14) | 0.872 | 1.02 (0.90, 1.15) | 0.784 |
| **Group 3 (ED to ED or INPT)** | |  |  |  |  |  |
| Overall | 1719/5545 | 31.0% | 1.02 (1.00, 1.03) | 0.036 | 1.02 (1.00, 1.03) | 0.009 |
| Deteriorating | 662/2184 | 30.3% | 1.03 (1.02, 1.05) | < 0.001 | 1.04 (1.02, 1.05) | < 0.001 |
| Improving | 1057/3361 | 31.4% | 0.92 (0.89, 0.95) | < 0.001 | 0.93 (0.90, 0.96) | <.0001 |
| **Group 4 (INPT to INPT)** | |  |  |  |  |  |
| Overall | 1563/3164 | 49.4% | 1.01 (1.00, 1.02) | 0.051 | 1.01 (1.00, 1.02) | 0.081 |
| Deteriorating | 937/1736 | 54.0% | 1.02 (1.00, 1.03) | 0.012 | 1.02 (1.00, 1.03) | 0.021 |
| Improving | 626/1428 | 43.8% | 0.97 (0.92, 1.03) | 0.304 | 0.97 (0.92, 1.02) | 0.254 |

**New Model 3 of Sensitivity Analysis:** Further adjusted for time intervals based on original Model 3.

**Supplementary Table 7.** Sensitivity analysis of 3-year all-cause mortality according to every 0.1 mg/dL change in S-Cre levels repeated within 24 hours after additionally adjusted for time intervals. ED, emergency department; INPT, inpatient; OPT, outpatient; S-Cre, serum creatinine.

|  |  |  | **Original Model 3** |  | **Further adjusted for time intervals between S-Cre measurements** |  |
| --- | --- | --- | --- | --- | --- | --- |
|  | **Case/N** | **Mortality (%)** | **Adjusted HR (95% CI)** | ***p*-value** | **Adjusted HR (95% CI)** | ***p*-value** |
| **3-year mortality** |  |  |  |  |  |  |
| Overall | 4166/14912 | 27.9% | 1.00 (0.99, 1.01) | 0.861 | 0.99 (0.98, 1.01) | 0.407 |
| Deteriorating | 2021/6753 | 29.9% | 1.06 (1.04, 1.08) | < 0.001 | 1.05 (1.03, 1.07) | < 0.001 |
| Improving | 2145/8159 | 26.3% | 0.96 (0.94, 0.98) | < 0.001 | 0.95 (0.93, 0.97) | < 0.001 |
| **Group 1 (OPT to OPT)** | |  |  |  |  |  |
| Overall | 423/4145 | 10.2% | 1.04 (0.9, 1.21) | 0.603 | 1.05 (0.91, 1.22) | 0.505 |
| Deteriorating | 215/1882 | 11.4% | 1.13 (0.94, 1.35) | 0.196 | 1.12 (0.93, 1.35) | 0.236 |
| Improving | 208/2263 | 9.2% | 0.94 (0.72, 1.23) | 0.654 | 0.96 (0.73, 1.26) | 0.751 |
| **Group 2 (OPT to ED or INPT)** | |  |  |  |  |  |
| Overall | 410/1761 | 23.3% | 0.96 (0.89, 1.04) | 0.347 | 0.96 (0.89, 1.04) | 0.351 |
| Deteriorating | 176/782 | 22.5% | 1.07 (0.95, 1.19) | 0.26 | 1.07 (0.95, 1.2) | 0.260 |
| Improving | 234/979 | 23.9% | 0.84 (0.73, 0.98) | 0.025 | 0.85 (0.73, 0.98) | 0.029 |
| **Group 3 (ED to ED or INPT)** | |  |  |  |  |  |
| Overall | 1719/5545 | 31.0% | 0.98 (0.96, 1.00) | 0.053 | 0.99 (0.97, 1.00) | 0.121 |
| Deteriorating | 662/2184 | 30.3% | 1.05 (1.02, 1.09) | < 0.001 | 1.06 (1.03, 1.1) | < 0.001 |
| Improving | 1057/3361 | 31.4% | 0.94 (0.92, 0.97) | < 0.001 | 0.95 (0.92, 0.97) | < 0.001 |
| **Group 4 (INPT to INPT)** | |  |  |  |  |  |
| Overall | 1563/3164 | 49.4% | 1.01 (0.99, 1.03) | 0.258 | 1.01 (0.98, 1.03) | 0.551 |
| Deteriorating | 937/1736 | 54.0% | 1.05 (1.03, 1.08) | < 0.001 | 1.04 (1.02, 1.07) | 0.002 |
| Improving | 626/1428 | 43.8% | 0.94 (0.90, 0.99) | 0.009 | 0.94 (0.89, 0.98) | 0.007 |

**New Model 3 of Sensitivity Analysis:** Further adjusted for time intervals based on original Model 3.

**Supplementary Table 8.** Summary of cut-off determination of ΔS-Cre% and ΔS-Cre for 30-day, 1-year, and 3-year all-cause mortality by patients’ service transition patterns and baseline status of impaired kidney function (IKF)**.** Numbers in bold represent significant effects of ΔS-Cre% or ΔS-Cre levels on all-cause mortality. ΔS-Cre, difference between baseline and second serum creatinine; ΔS-Cre%, percent change between baseline and second serum creatinine.

|  | **1-year** | | **30-day** | |  | **3-year** | | | |
| --- | --- | --- | --- | --- | --- | --- | --- | --- | --- |
|  | **Group 1** | **Group 2** | **Group 3** | **Group 4** |  | **Group 1** | **Group 2** | **Group 3** | **Group 4** |
| **ΔS-Cre%** |  |  |  |  |  |  |  |  |  |
| **IKF positive** |  |  |  |  |  |  |  |  |  |
| Deteriorating | 3.06% | 1.64% | 10.26% | **13.00%** |  | 3.26% | **1.64%** | 6.21% | 6.67% |
| Improving | -3.65% | **-3.57%** | -10.11% | -4.91% |  | -2.58% | **-3.57%** | **-18.09%** | -10.10% |
| **IKF negative** |  |  |  |  |  |  |  |  |  |
| Deteriorating | 5.81% | 20.83% | **22.76%** | **20.27%** |  | 2.98% | 20.00% | **17.86%** | **20.34%** |
| Improving | -10.98% | **-3.81%** | -20.00% | -13.33% |  | -10.98% | **-4.65%** | **-23.02%** | -3.49% |
| **ΔS-Cre (mg/dL)** |  |  |  |  |  |  |  |  |  |
| **IKF positive** |  |  |  |  |  |  |  |  |  |
| Deteriorating | 0.09 | 0.33 | 0.10 | **0.22** |  | 0.06 | 0.33 | 0.10 | **0.22** |
| Improving | -0.11 | **-0.10** | -0.11 | -0.37 |  | -0.06 | **-0.12** | **-0.40** | **-0.20** |
| **IKF negative** |  |  |  |  |  |  |  |  |  |
| Deteriorating | 0.04 | 0.14 | **0.16** | **0.12** |  | 0.04 | 0.14 | **0.15** | **0.10** |
| Improving | -0.08 | **-0.05** | -0.20 | -0.10 |  | -0.01 | **-0.10** | **-0.20** | -0.04 |

**Supplementary Figure 1.** Difference in S-Cre levels repeated within 24 hours (within-day ΔS-Cre) over sampling time interval by patients’ service transition patterns. Red line: baseline IKF positive; blue line: baseline IKF negative. IKF, impaired kidney function; S-Cre, serum creatinine.


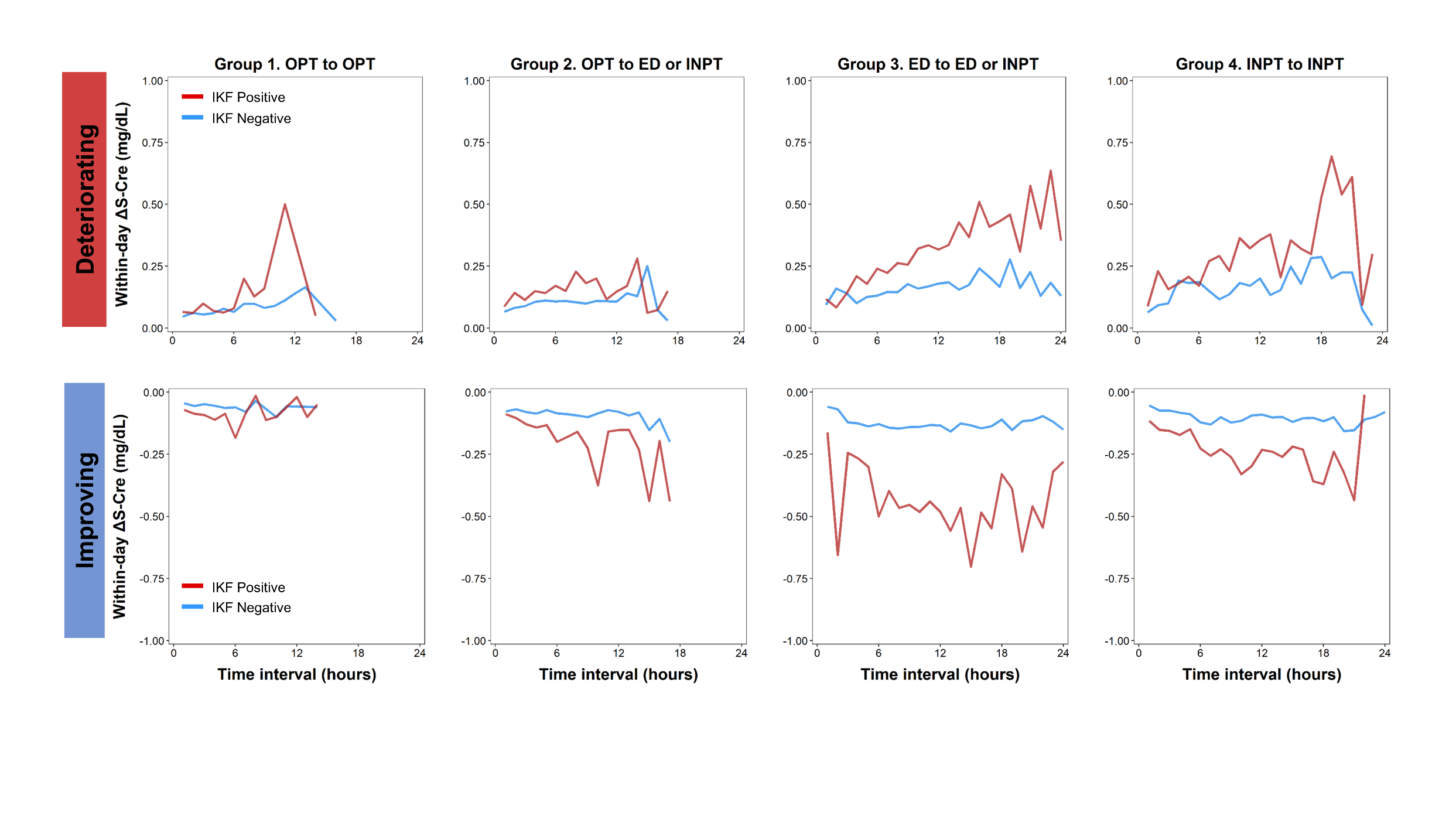


**Supplementary Figure 2.** Adjusted hazard ratios (aHRs) for 30-day (red line), 1-year (dark-red line), and 3-year (blue line) all-cause mortality according to the difference in S-Cre levels repeated within 24 hours (within-day ΔS-Cre) by patients’ service transition patterns and variation directions (deteriorating vs. improving). Solid lines represent aHRs based on restricted cubic splines for within-day ΔS-Cre, with knots at the 5^th^, 25^th^, 50^th^, 75^th^, and 95^th^ percentiles. Shaded areas represent the upper and lower 95% confidence intervals. Reference was set at 10th percentile of ΔS-Cre levels. Variables adjusted are the same as that shown in Model 3 of Table 3. S-Cre, serum creatinine.

**
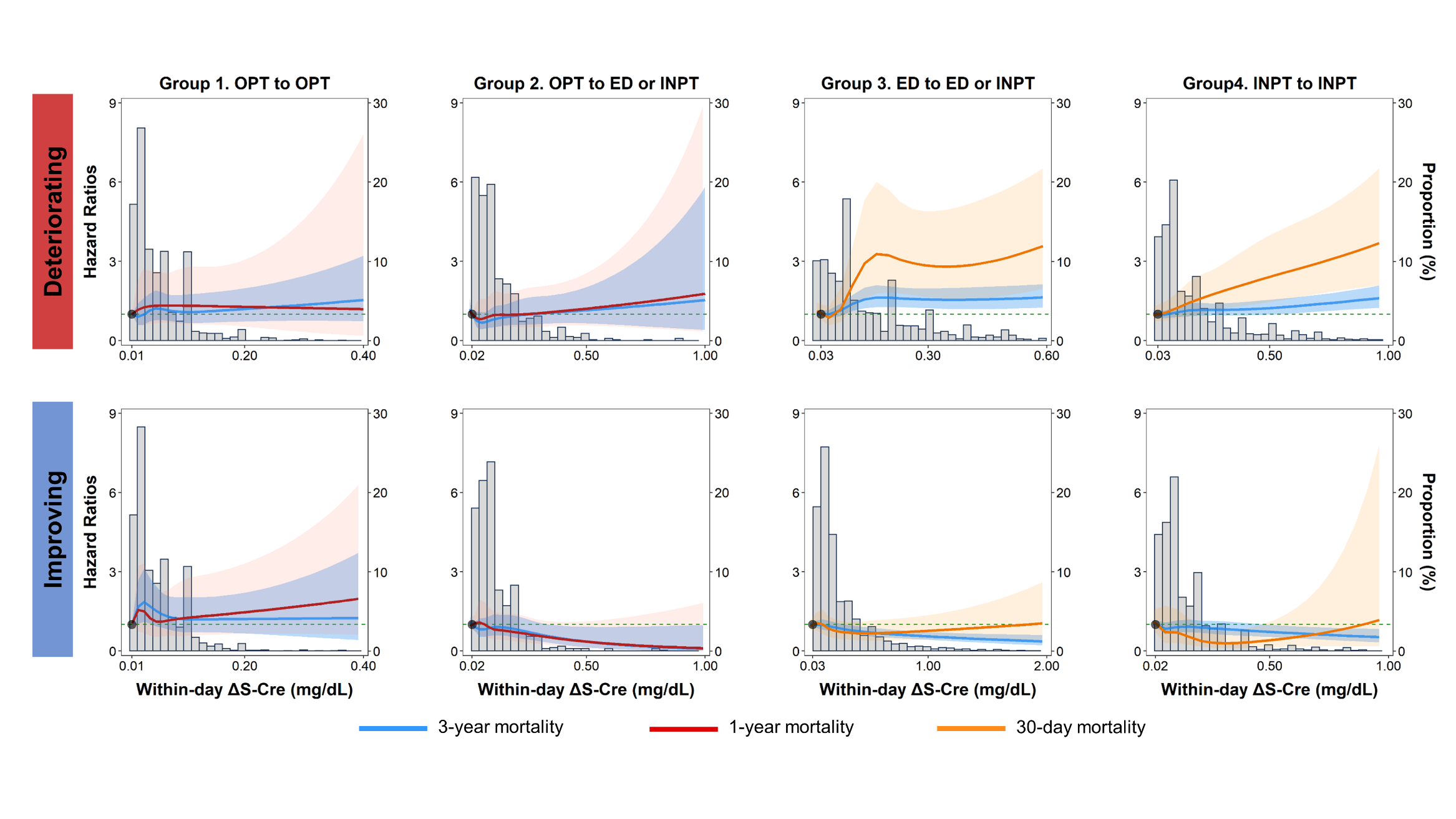
**

**Supplementary Figure 3.** Cut-off determination of ΔS-Cre% for 30-day and 1-year all-cause mortality by patients’ service transition patterns**.** Dots in black and gray represent significant and nonsignificant effects of ΔS-Cre% levels on all-cause mortality, respectively, shown in Model 3 of Table 3. ΔS-Cre%, percent change between baseline and second serum creatinine.


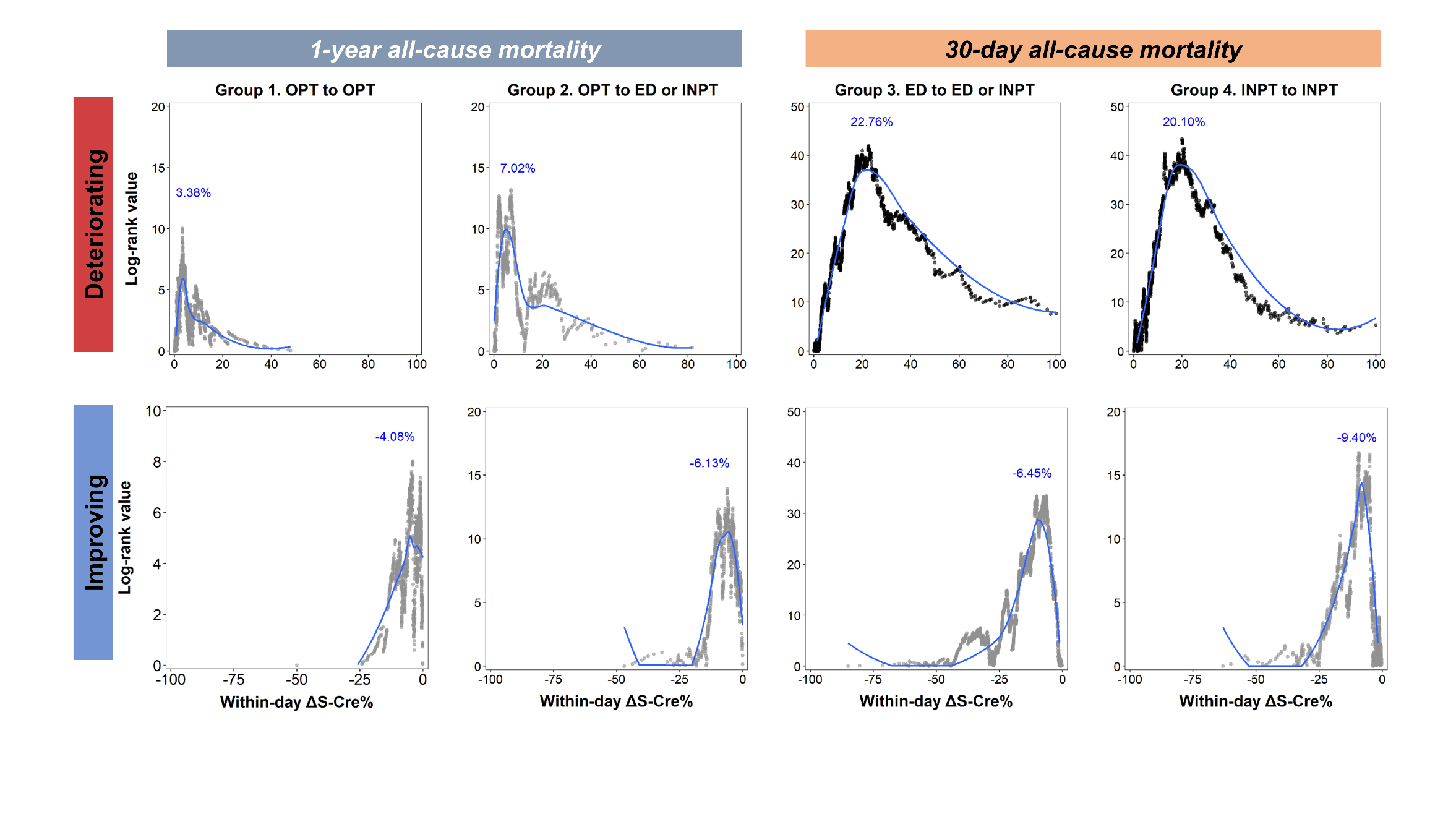


**Supplementary Figure 4.** Cut-off determination of ΔS-Cre for 30-day and 1-year all-cause mortality by patients’ service transition patterns**.** Dots in black and gray represent significant and nonsignificant effects of ΔS-Cre levels on all-cause mortality, respectively, shown in Model 3 of Table 3. ΔS-Cre, difference between baseline and second serum creatinine.

**
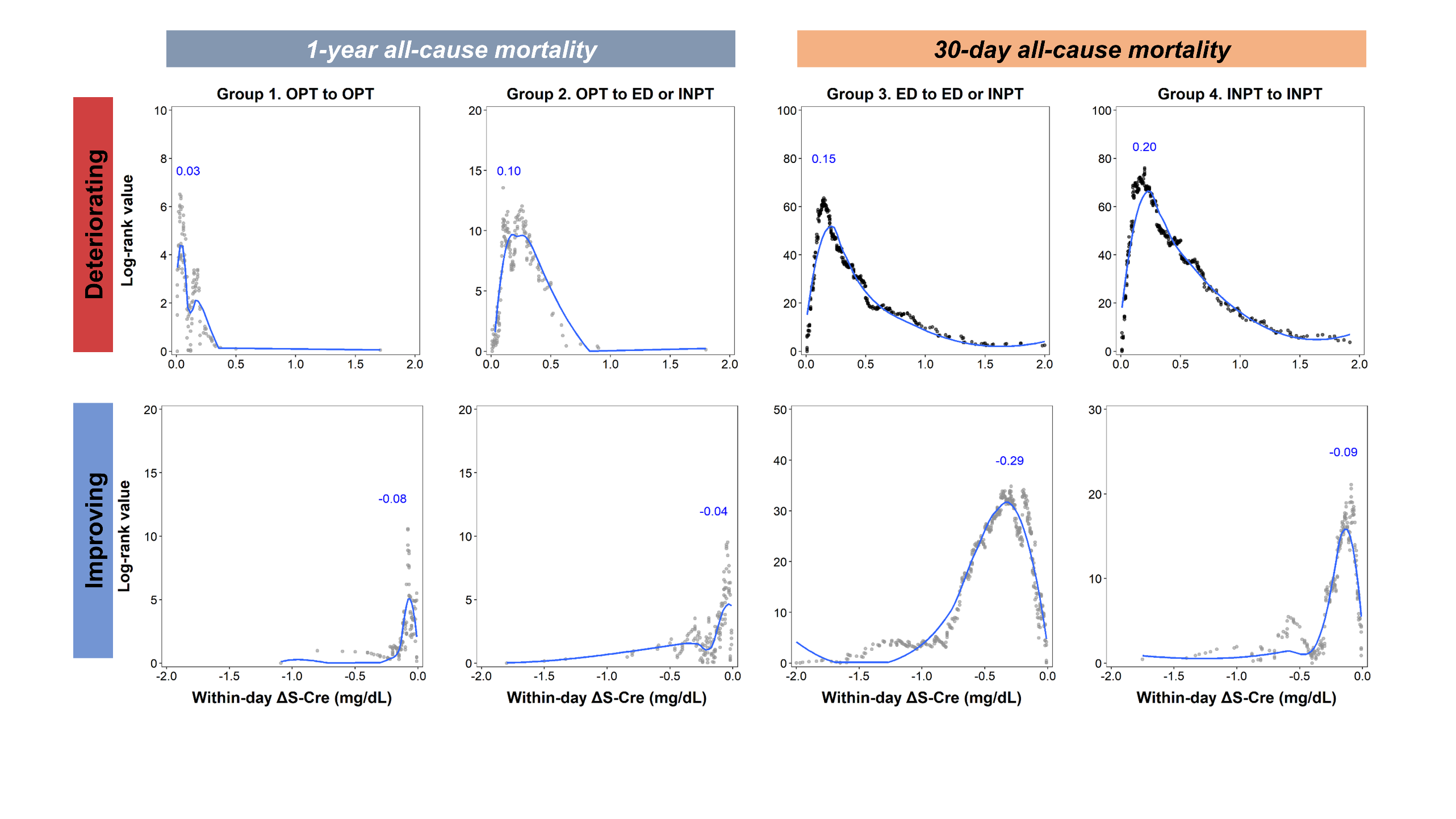
**

**Supplementary Figure 5.** Study flow chart of 14912 patients who were included in analysis. ESRD, end stage renal disease; PD, peritoneal dialysis; HD, hemodialysis; CVVH, continuous venovenous hemofiltration; DFPP, double filtration plasmapheresis; APD, automated peritoneal dialysis; CPCR, cardiopulmonary-cerebral resuscitation; S-Cre, serum creatinine; OPT, outpatient; ED, emergency department; INPT, inpatient.

**
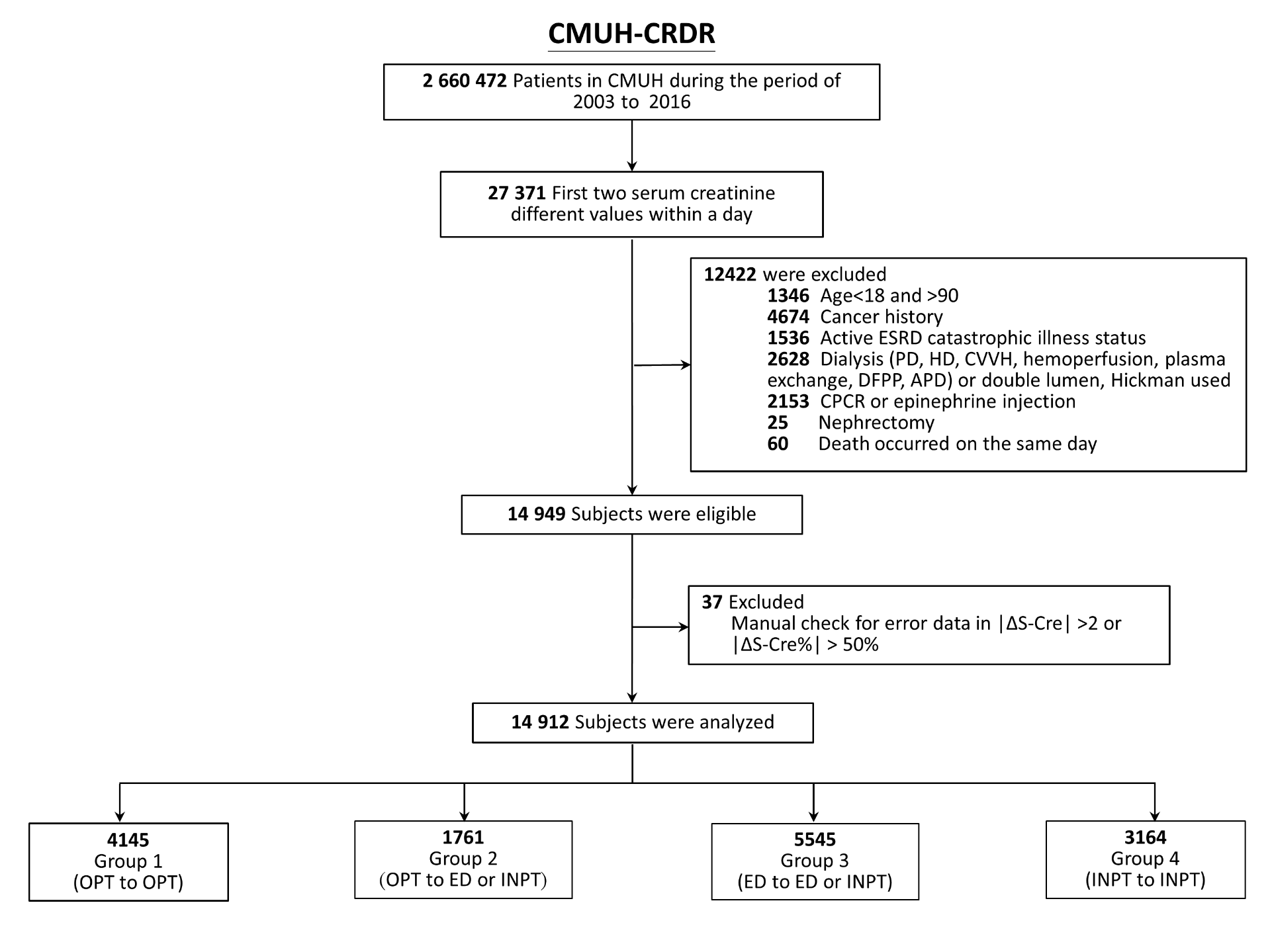
**
